# Automated Universal BRAF State Detection within the Activation Segment in Skin Metastases by Pyrosequencing-Based Assay U-BRAF^V600^

**DOI:** 10.1371/journal.pone.0059221

**Published:** 2013-03-26

**Authors:** Alexander Skorokhod, Peter Helmbold, Benedikt Brors, Peter Schirmacher, Alexander Enk, Roland Penzel

**Affiliations:** 1 Department of Dermatology, Heidelberg University Hospital, Heidelberg, Germany; 2 Department of Theoretical Bioinformatics, German Cancer Research Center (DKFZ), Heidelberg, Germany; 3 Institute of Pathology, Heidelberg University Hospital, Heidelberg, Germany; The University of Queensland, Australia

## Abstract

Malignant melanoma is a highly-aggressive type of malignancy with considerable metastatic potential and frequent resistance to cytotoxic agents. BRAF mutant protein was recently recognized as therapeutic target in metastatic melanoma. We present a newly-developed U-BRAF^V600^ approach – a universal pyrosequencing-based assay for mutation detection within activation segment in exon 15 of human *braf*. We identified 5 different BRAF mutations in a single assay analyzing 75 different formalin-fixed paraffin-embedded (FFPE) samples of cutaneous melanoma metastases from 29 patients. We found BRAF mutations in 21 of 29 metastases. All mutant variants were quantitatively detectable by the newly-developed U-BRAF^V600^ assay. These results were confirmed by ultra-deep-sequencing validation (^∼^60,000-fold coverage). In contrast to all other BRAF state detection methods, the U-BRAF^V600^ assay is capable of automated quantitative identification of at least 36 previously-published BRAF mutations. Under the precaution of a minimum of 3% mutated cells in front of a background of wild type cells, U-BRAFV600 assay design completely excludes false wild-type results. The corresponding algorithm for classification of BRAF-mutated variants is provided. The single-reaction assay and data analysis automation makes our approach suitable for the assessment of large clinical sample sizes. Therefore, we suggest U-BRAF^V600^ assay as a most powerful sequencing-based diagnostic tool to automatically identify BRAF state as a prerequisite to targeted therapy.

## Introduction

Malignant melanoma is a highly-aggressive skin cancer and one of the most metastatic malignancies [1]. Studies revealed that regulation of the Ras-Raf-MAPK pathway is abrogated in the majority of melanoma tumors as a result of activating NRAS or BRAF mutations, which are mutually exclusive and present in up to 90% of cutaneous melanomas [2]. BRAF mutation is an early event in tumorigenesis: it may already occur in benign nevus, but by itself, it is not sufficient to induce cancer [3]. It was suggested that effects of NRAS and BRAF mutations may be limited to early disease stages and that other factors are more relevant after regional metastases have occurred [4]. Somatic mutations in the BRAF oncogene have been documented with high frequency in cutaneous melanomas, occurring in 50 to 70% of tumor samples [5]. BRAF mutations are also found in 40 to 70% of papillary or anaplastic thyroid cancers and in small percentages of many other types of tumor [6]. Most BRAF mutations occur at codon V600 and constitutively activate BRAF together with the corresponding downstream signal transduction in the MAP kinase pathway [6]. This mutation significantly increases the risk of mortality both in colorectal cancer patients and in patients with malignant melanoma [7].

The BRAF kinase inhibitor vemurafenib, recently approved by the Food and Drug Administration (FDA), represents significant progress in melanoma therapy: patients’ treatment with vemurafenib resulted in complete or partial tumor regression in the majority of patients with BRAF^V600E^-positive metastatic melanoma [8].

Current report presents a U-BRAF^V600^ approach that enables automated BRAF mutation detection within the activation segment in exon 15 by a single pyrosequencing-based assay.

## Methods

### Ethics Statement

The study was approved by the Institutional Review Board of the Heidelberg University Hospital, Germany, and all patients signed written informed consent at time of initial clinical investigation.

### FFPE Tissue Samples and Cell Lines

Formalin-fixed paraffin embedded (FFPE) tissue cutaneous metastasis samples were examined in this study. Diagnoses were independently established and controlled in each tumoral sample according to histopathological standards by two experienced dermopathologists (P.H., co-author, and Wolfgang Hartschuh, Department of Dermatology, University of Heidelberg).

A549 cells and wild-type HeLa cell lines were purchased from the ATCC (American Type Culture Collection).

### DNA Extraction and Pyrosequencing

For the analysis of tumor samples, haemoltoxylin- and eosin-stained slides were reviewed by an experienced pathologist (P.H., co-author) to ensure sufficient viable tumor content (60–90% tumor cells).

Total genomic DNA was extracted from seven 10 µm-thick unstained sections of FFPE tissue blocks according to manufacturer’s instructions, using an automated DNA Extractor (Qiasymphony™, Qiagen). To avoid cross-contamination, a new disposable microtome blade was used for each FFPE tissue block. In addition, knife holder and anti-roll plate was wiped down with 100% ethanol in between each block. The total DNA was eluted in 50 µl and immediately stored at −20°C for later use. The eluted DNA was quantified using a Qubit dsDNA HS Assay (Invitrogen).

For pyrosequencing assay, the region of human *braf* spanning mutation sites within the activation segment in exon 15 was amplified using forward primer U-BRAF-F and biotinylated reverse primer BRAF-Pyro-R (Eurofins MWG Operon, **Table S1 in File S1**). Each PCR reaction mixture was prepared with 2–10 ng genomic DNA, 5 pmol each primer, 2.5 mM dNTPs and 1 unit Phusion™ polymerase (Biozym) in a total volume of 50 µl. Amplification of BRAF fragment was performed in a PCR cycler Flexcycler (Analytik Jena) as follows: 98°C for 1 minute, 35 cycles of 98°C for 10 seconds, 56°C for 20 seconds and 72°C for 20 seconds, followed by final extension at 72°C for 10 minutes. Specific amplification of the 229-bp fragment was verified by visualizing 5 µl PCR product on a 2% agarose TBE gel using SubCell electrophoresis unit (Bio-RAD), followed by 30-minute incubation in 1^x^ GelRed solution (Biotium).

Pyrosequencing procedure was performed identifying variant mutations either at codons V600 to S602 (5′-AGTGAAATCT-3′) with sequencing primer U-BRAF-600-Seq or at codons T599 to S602 (5′-TACAGTGAAATCT-3′) with sequencing primer U-BRAF-599-Seq (Eurofins MWG, **Table S1 in File S1**). 20 µl PCR product (400–500 ng) were used for pyrosequencing according to manufacturer’s instructions (Pyromark Q24, Qiagen). Sequence pyrograms were automatically analyzed using simple operators of a spreadsheet application.

### Sanger Sequencing

Sanger sequencing was performed bidirectionally with 1 µl PCR product amplified for pyrosequencing as described above, using BRAF-15F-Seq and BRAF-15R-Seq (Eurofins MWG Operon, **Table S1 in File S1**) with Big Dye Terminator V1.1 cycle sequencing reagents (Life Technologies) under the following PCR conditions: 25 cycles at 95°C for 20 seconds, 55°C for 15 seconds, and 60°C for 1 min. DNA sequences were finally determined on a 3500 Gene Analyzer (Life Technologies) and each sample was visually analyzed for the presence of mutation of *braf* within activation segment in exon 15.

### Cloning of BRAF Mutant Variants

Samples with p.V600E, p.V600E2, p.V600K, p.VKS600_602>DT or p.V600E;K601I mutations were amplified using U-BRAF-F and BRAF-Pyro-R as described above. After purification according to manufacturer’s instructions (QIAquick PCR Purification kit, Qiagen), the amplified products were incubated with 1 Unit Taq polymerase in the presence of 0.2 mM ATP for 30 min at 72°C. The purified PCR products were ligated into pSTBlue-1 vector, followed by transformation into XL1-Blue competent cells according to manufacturer’s instructions (AccepTor® Vector kit, Merck). The clones were selected by PCR amplification of a single colony using U-BRAF-F and biotinylated BRAF-Pyro-R (**Table S1 in File S1**). The mutations, as well as the wild type, were confirmed by U-BRAF pyrosequencing using sequencing primer U-BRAF-599-Seq (**Table S1 in File S1**). Plasmids were isolated according to manufacturer’s instructions (Plasmid Isolation kit, Roche). Plasmid DNA was quantified using a Qubit dsDNA HS Assay (Invitrogen).

### cobas 4800 BRAF V600 Mutation Test Analysis

Total genomic DNA was extracted from seven 10 µm-thick unstained sections of FFPE tissue blocks according to manufacturer’s instructions (cobas DNA Sample Preparation Kit, Roche). The extracted DNA was quantified using a Qubit dsDNA HS Assay (Invitrogen). Samples, containing at least 125 ng DNA in 25 µl, were subjected to cobas® 4800 BRAF V600 Test assay according to manufacturer’s instructions (Roche). The results were reported as “Mutation Detected”, “Mutation Not Detected” or “Invalid”.

### MiSeq Ultra-Deep Sequencing and Biostatistical Analysis

Based on MiSeq technology (Illumina), the two-round PCR strategy was designed for ultra-deep-sequencing analysis, integrating Ullimina’s Universal Adapter and TruSeq Adapter into amplified fragments containing complete exon 15 of *braf*. 1^st^ round PCR was performed with primers MiSeq-Rev and individually for each sample MiSeq-Fxx (Eurofins MWG Operon) using 1 unit Phusion™ polymerase. To facilitate the demultiplexing in one assay, the in-line indices (barcodes) from 4-bp to 8-bp were integrated into MiSeq-Fxx primers (**Table S1 in File S1**) 5 µl PCR product was cleaned using ExoSAP-IT reagent according to manufacturer’s instructions (Affymetrix).

2^nd^ round PCR was performed on 1 µl purified PCR product using 5 pmol Ullimina’s Universal Adapter and TruSeq Adapter primers in 50 µl total (**Table S1 in File S1**). PCR conditions for both rounds were as follows: 98°C for 1 minute, 25 cycles of 98°C for 10 seconds, 56°C for 20 seconds and 72°C for 20 seconds, followed by final extension at 72°C for 10 minutes. Specific amplification of fragments from 280-bp to 284-bp was verified by visualizing 5 µl PCR product on a 2% agarose TBE gel using a SubCell electrophoresis unit (Bio-RAD), followed by 30-minute incubation in 1^x^ GelRed solution (Biotium). PCR products were purified according to manufacturer’s instructions (QIAquick PCR Purification kit, Qiagen). DNA concentration was quantified using HS Assay with Qubit dsDNA HS Assay (Invitrogen).

For MiSeq analysis, all amplified fragments were pooled into a 10 nM library. MiSeq assay yielded output data in FASTQ-format, which were subjected to sequence quality analysis using fast length adjustment of short reads (F.L.A.Sh) [9]. The obtained data file was split into individual FASTQ-files according to integrated in-line barcodes using FASTAX barcode splitter script (Version 0.0.13.2). FASTQ files were aligned against the hg19 reference sequence with Burrows-Wheeler Aligner (BWA, Version 0.5.9-r16) and standard parameter settings. Variants were called from the resulting BAM files using SAMtools/BCFtools (Version 0.1.17) as integrated into an in-house pipeline [10]. Briefly, only reads with a minimum mapping quality of 30 and bases with minimum base quality of 13 (phred score) were considered. Bases at each position were obtained by SAMtools Mpileup, and BCFtools was applied with changed prior probability to account for allele frequencies strongly deviating from 0.5 or 1.0. Additional filters were employed to remove false positive calls, requiring at least two reads to support a variant, and removing variants due to typical Illumina sequencing artifacts [11].

## Results

We analyzed BRAF state in 75 formalin-fixed paraffin-embedded (FFPE) samples of cutaneous melanoma metastases from 29 patients (age 62±25, male-to-female ratio 1.9). By Sanger sequencing, we identified five different types of BRAF mutations reported by our group previously [12] in 18 of 29 patients (62%, **Table 1**).

**Table 1:** BRAF mutations within activation segment in exon 15 in cutaneous melanoma metastases.

Case	Sample	Age/Sex	Sanger sequencing	Pyrosequencing mt:wtratio in % 1	Deep-Sequencing mt:wtratio in % 1	cobas^2^
1	A,B	53/f	V600E	24; 25	21; 21	+
2	A,B	47/f	V600E	25; 26	19; 22	+
3	A,B,C	40/m	V600E	16; 20; 20	9; 15; 14	+
4	A,B	79/m	V600E	53; 59	52; 60	
5	A	55/m	–	–	–	
6	A,B,C	69/m	–	–	–	–
7	A	80/m	V600E	33	33	
8	A,B,C,D,E	53/f	V600E	36; 18; 26; 25; 35	35; 14; 20; 22; 35	
9	A	87/f	–	–	2^V600E^	
	B				–	
10	A,C,B	80/m	V600E	56; 62; 45	55; 62; 43	
11	A,B,C	82/f	–	–	1; 1; 1^V600E^	–
12	A	83/m	–	–	–	
13	A,B,C,D	56/m	–	–	–	
14	A,B,C,D,E	57/m	VKS600_602>DT	33; 23; 37; 24; 35	33; 22; 38; 22; 37	
15	A	74/f	–	9^V600E^	4^V600E^	–
	B			–	–	
16	A,B	65/m	V600E	21; 24	11; 17	
17	A	52/m	–	10	7	–
	B		V600K	17	16	
18	A	30/m	V600E	28	24	
19	A	75/f	–	11^V600E^	5^V600E^	–
20	D	66/m	V600E	22	14	
	A,B,C,E		–	17; 13; 13; 11	7; 6; 6; 5	–
21	A,B	73/m	V600E2	27; 34	31; 36	–
22	A,B	37/m	V600K	39; 39	44; 43	
23	A	71/f	–	–	–	
24	A,B	52/m	–	20; 9^V600E^	9; 3^V600E^	
25	A	54/f	V600E	16	11	
26	A,B,C,D,E,F	66/m	–	–	–	
	G,H				2; 2^V600E^	
27	A,B,C,D,E	54/m	V600K	49; 43; 47; 42; 56	49; 45; 46; 47; 61	+
28	A,B	78/f	V600E	21; 26	9; 12	
29	A,B	44/m	V600E; K601I	61; 39	61; 40	–

different samples of the same tumor are specified by 1, 2 etc., different tumors of the same patient specified by A, B etc.; age in years, f = female, m = male;

1 wt – wild type, mt - mutant.

2 “+” Mutation Detected, “–” Mutation Not Detected (cobas® 4800 report).

### Novel Pyrosequencing-based U-BRAF^V600^ Assay

To prove these data, we performed the pyrosequencing analysis with the conventional dispensation order G1A2C3G4[A5T6]G7A8T9 generated by Pyromark Q24 software Version 2.0.6 (Qiagen) flanking the hotspot mutation T1799A at codon V600 and ending with the first nucleotide of codon S602. Negative nucleotide dispensations G1 and C3 were included as internal controls. Although T1799A mutation was determined by this dispensation order, the variant mutations beyond V600E resulted in unsolved aberrant pyrograms (**Figure S1a**).

**Figure 1:**
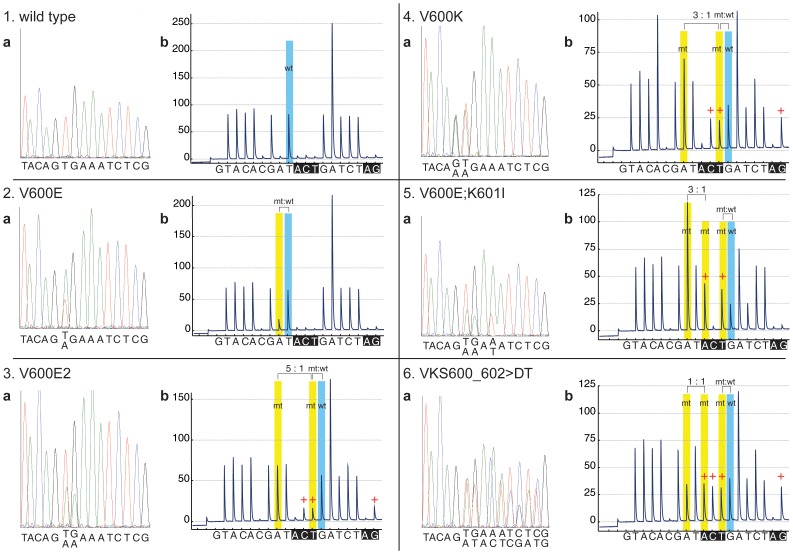
BRAF mutation analysis by Sanger sequencing and pyrosequencing-based assay U-BRAF^V600^. (**a**) Sanger sequencing; (**b**) pyrosequencing-based assay U-BRAF^V600^. “+” indicates the positive peaks of the dispensation nucleotides within recognition patterns of U-BRAF^V600^ assay. mt – mutant; wt – wild-type. Recognition patterns are shown in black boxes.

**Figure 2:**
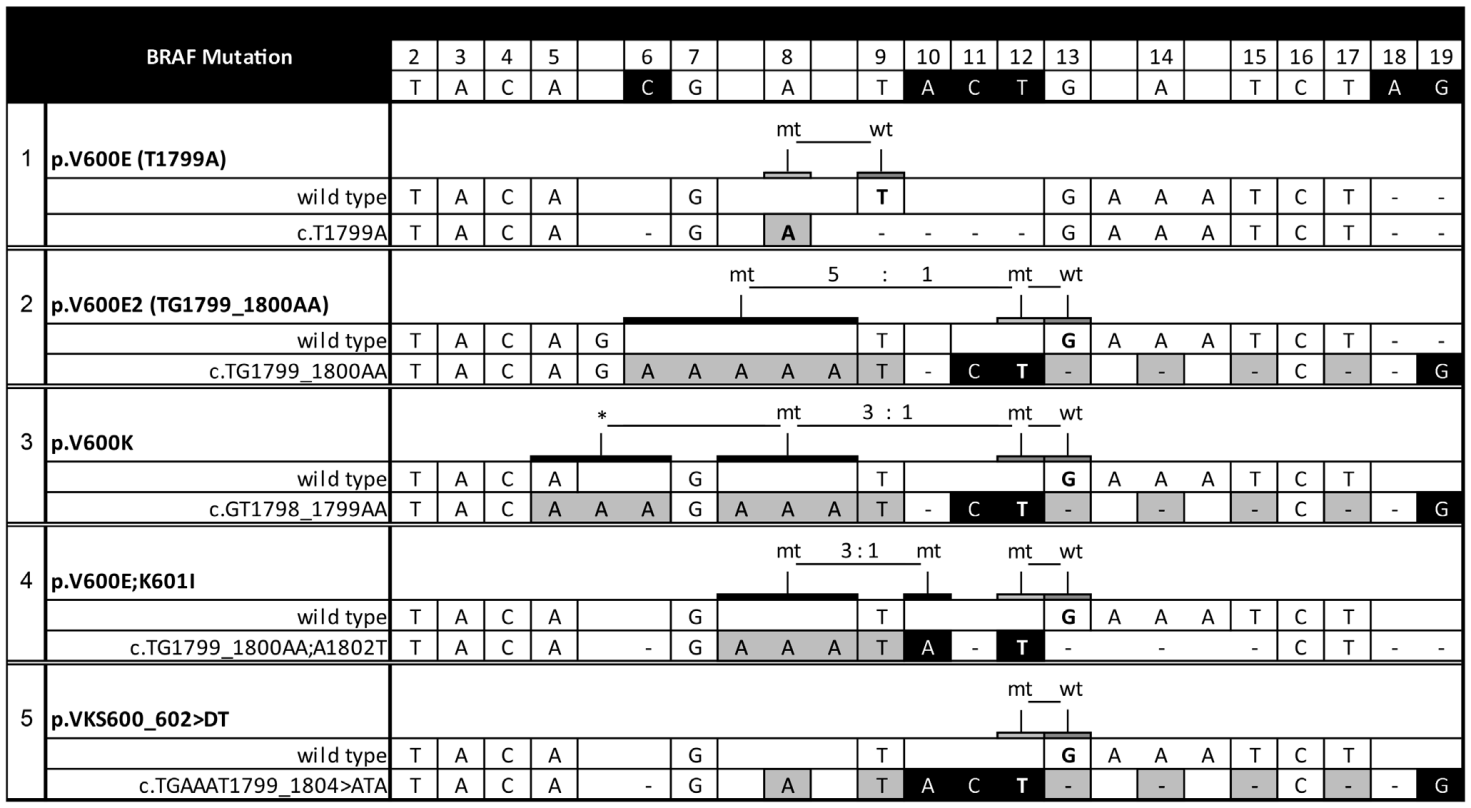
Low-abundance BRAF mutations. **a**) Pyrogram of cloned wild-type BRAF. Red arrow indicates the reduction of peak intensity values; **b**) pyrograms of cloned BRAF mutants. Red asterisks indicate the dispensation nucleotide’s peaks, which are characteristic for corresponding BRAF mutant in low-copy-number analysis; **c**) pyrograms of premixed BRAF mutants with wild type. Red arrows indicate the tendency of peak-pairs’ difference included in low-copy-number analysis. Red asterisks indicate the peaks with the contribution of correspondent mutant nucleotides shown in (**b**).

To overcome this limitation, we designed the novel dispensation order U-BRAF^V600^– G1T2A3C4A5C6G7A8T9[A10C11T12]G13A14T15C16T17[A18G19]. Because the knowledge of specific variant in each case could explain the altered pyrogram tracing created by a change in order and/or quantity of incorporation of each nucleotide, we embedded the two recognition patterns [A10C11T12] and [A18G19], enabling the simultaneous identification of hotspot V600E mutation together with variant mutations with two-nucleotide substitutions p.V600E2 (c.TG1799_1800AA) and p.V600K (c.GT1798_1799AA), tandem mutation p.V600E;K601I (c.TG1799_1800AA;A1802T) and complex in-frame mutation VKS600_602>DT (c.TGAAAT1799_1804>ATA) [12]. Here, the presence of variant mutations affects the pyrogram sequence pattern by re-distribution of nucleotide incorporation in the mutant DNA sequence, resulting in a unique pyrogram for each BRAF mutation (**Figure 1**). Both recognition patterns differentiate the individual mutations by the presence of the corresponding peaks characteristic for each mutation variant. Furthermore, the ratio A8:T12 distinguishes between mutations V600E2 (5∶1) and V600K (3∶1) (**Figure 2**).

**Figure 3:**
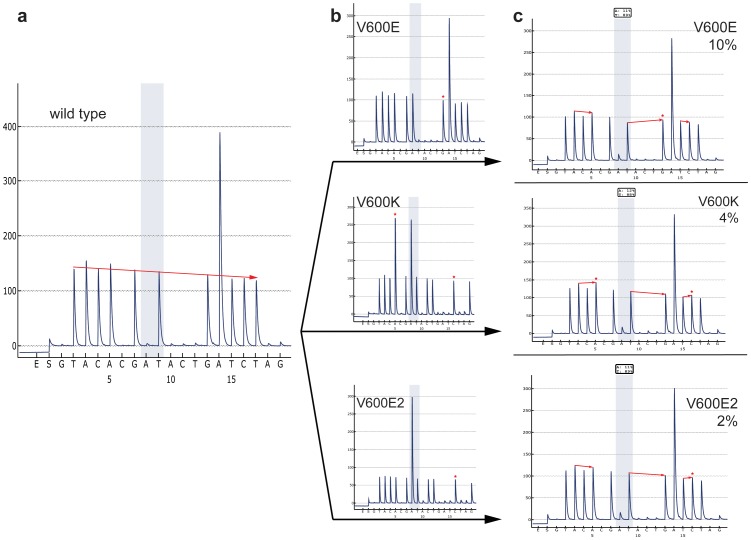
Dispensation order for 5 mutated BRAF variants detected by U-BRAF^V600^ assay. *A5 = Awt +3Amt. Recognition patters are indicated in black boxes, individual mutation features are marked in grey boxes dispensation order’s nucleotides, which are involved into mt:wt ratio, are bolded.

We found that at least 400 ng PCR product is required for successful analysis by U-BRAF^V600^ assay, although in this case the signal intensity is constantly reduced by each dispensation step (**Figure 3a**). In our study, up to 1% reduction was observed per dispensation step from the initial intensity value of dispensation nucleotide T2 resulting in formula [“reduction factor”×N]%, where “N” is dispensation nucleotide’s number. Therefore, this reduction factor should be taken into consideration in calculating both mutant-to-wild-type ratio and reference peaks’ intensities.

Sequence pyrograms were automatically analyzed using logical operator “IF” in spreadsheet application (**Table S2 in File S1**). Wild-type-threshold was determined according to A8:T9 ratio of wild-type reference controls A549 and wild-type HeLa cell lines. Comparing with Sanger sequencing data, three more cases were identified as BRAF mutants (**Table 1**). Moreover, samples of cases 17 and 29, which were only detected in part by Sanger sequencing, were all determined as mutant-positive by U-BRAF^V600^ analysis (**Table 1**). These data demonstrate the higher sensitivity of pyrosequencing assay resulting in 21 BRAF-mutated cases of 29 cutaneous metastases (72.4%).

### Cases with Low-abundance BRAF Mutation

In case of low-copy-number BRAF- mutated samples (5% or less), the recognition patterns can be masked by background noise and, therefore, pyrograms of V600K, V600E2 or V600E;K601I could be very difficult to distinguish from V600E mutation in analyzing only the conventional A8:T9 ratio. To simulate low-abundance BRAF mutation templates, we subcloned these mutant variants as well as wild type *braf* exon 15. The clones containing V600E, V600E2 or V600K were individually mixed together with the plasmid, containing wild-type *braf*, in a proportion from 1% to 10% mutant variant and subjected to PCR amplification followed by U-BRAF^V600^ pyrosequencing. Analyzing only the A8:T9 ratio, 2% V600E2 can be misinterpreted either as 10% V600E or as 4% V600K (**Figure 3c**). In this case, the ratios A3:A5, T9:G13 and T15:C16 should be taken into consideration in estimating the mutant-specific portion in signal intensities of A5, G13 or C16 (**Figure 3b**). In general, the presence of variant mutations beyond V600E can be determined by the difference in peak intensity values in comparison with correspondent wild-type reference peaks (**Figure 2** **,** **Figure 3c**). Importantly, G19 is prone to higher background noise (**Table S2 in File S1**) and should therefore be excluded from the low-abundance BRAF mutation analysis.

### MiSeq Ultra-deep Sequencing Validation of U-BRAF^V600^ Data

To prove both the sensitivity and the specificity of U-BRAF^V600^ assay, several FFPE samples, which yielded at least 125 ng DNA in 25 µl, were subjected to cobas® BRAF V600 Mutation Test assay. In our study, due to initially low biopsy amount, only a few FFPE samples were suitable to perform at least one cobas® BRAF V600 Mutation Test assay analysis. As expected, mutations p.V600E2 (case 21), p.V600E;K601I (case 29) and p.VKS600_602>DT (case 14) were not detected by cobas® BRAF V600 Mutation Test assay, whereas both p.V600E (cases 1, 2, 3) and p.V600K (case 27) were identified as V600-mutated cases.

Unfortunately, cases 15, 17, 19 and 20 with low-abundance V600E mutation were not detected by Sanger sequencing, and also not identified by cobas® 4800 BRAF V600 Mutation Test assay (**Table 1**). Therefore, the examined cases were further subjected to ultra-deep-sequencing analysis using MiSeq assay (Illumina). Ultra-deep sequencing of all 75 samples yielded typical coverage in the target region (exon 15 of *braf*) of 50,000 to 80,000-fold (Submission ID: SUB157783, Sequence Read Archive (SRA), NCBI BioSample Submissions). Sequence reads were aligned with Burrows-Wheeler Aligner against the hg19 reference sequence, and variants were called using an in-house pipeline based on SAMtools/BCFtools. Variant reads at positions indicative for the studied BRAF mutations were counted and variant allele frequencies were calculated. These calculations confirm the results of the pyrosequencing assay in all cases (**Table 1**). Interestingly, samples with low-abundance mutation level showed constantly higher mt:wt ratio in pyrosequencing data analysis in comparison with ultra-deep-sequencing assay. In addition, cases 9 and 26 were partially detected with 2% V600E, and case 11 with 1% V600E (**Table 1**).

## Discussion

Sanger (direct) sequencing is widely accepted as a gold standard routinely used to detect down to 20% BRAF mutation level in biopsy specimens [13]. Alternative approaches, like cobas® BRAF V600 Mutation Test (Roche) or BRAF RGQ PCR (Qiagen), claim to detect mutations down to 1.27% level in a wild-type background. Nevertheless, as quantitative PCR-based approaches, they have limited precision and present difficulties in reliably detecting low-copy-number templates due to nonspecific amplification and competitive side reactions [14]. Unfortunately, the FDA-approved cobas 4800 BRAF V600 Mutation Test is not able to distinguish between mutations V600E, V600K and V600E2. Moreover, according to the FDA’s Summary of Safety and Effectiveness Data (SSED), less than 30% V600K mutants and below 68% of V600E2 mutation (c.TG1799_1800AA) are not detectable by cobas BRAF V600 Mutation Test assay. BRAF mutation assays based on **r**estriction **f**ragment **l**ength **p**olymorphism analysis (RFLP) and **s**ingle-**s**trand **c**onformation **p**olymorphism analysis (SSCP) are less sensitive and less specific than Sanger sequencing [15].

In contrast, pyrosequencing, a real-time sequencing-by-synthesis approach, has a high throughput and is capable of detecting minor sequencing variants with greater diagnostic sensitivity than Sanger sequencing. It shows high accuracy and precision of pyrosequencing in quantitative identification of BRAF mutations in melanoma cell lines as well as in FFPE tumors [16]. Even though the approaches based on **s**hifted **t**ermination **a**ssay (STA) and **a**mplification **r**efractory **m**utations **s**ystem **a**llele-**s**pecific PCR (ARMS AS-PCR) give comparably sensitive results, they are still designed for detection of very few BRAF mutation variants. In general, to avoid false wild-type detection, Sanger sequencing is required for all available BRAF state detection methods in case of variant mutations beyond V600E/K/D/R/A.

A commercially-available pyrosequencing assay for BRAF state detection – therascreen® BRAF Pyro® Kit (Qiagen) – is designed to analyze the antisense strand of *braf* starting directly at codon V600. In this particular case, due to mismatching of sequencing primer, a sample with variant mutations downstream from codon V600 will be identified as a false wild-type. Moreover, V600K or V600R mutants may be interpreted as a false V600E mutation at mutant-to-wild-type ratio equal to 25% or less.

We designed a pyrosequencing assay U-BRAF^V600^ analyzing the sense strand of human *braf* within the activation segment in exon 15 towards the mutations, deletions and/or insertions, which affect the codons downstream from V600. Importantly, unique recognition patterns embedded into U-BRAF^V600^ make it possible to analyze all 5 different mutations in our study – both single- (p.V600E) and two-nucleotide substitutions (p.V600E2 and p.V600K), tandem mutation p.V600E;K601I as well as complex in-frame mutation p.VKS600_602>DT [12] – in one single assay. Moreover, compared with Sanger sequencing, where complex deletions and/or insertions require laborious manual analysis, the complex in-frame mutation p.VKS600_602>DT [12] was easily identified using binary (yes/no) data of recognition patterns (**Table S2 in File S1**).

We next asked whether our approach could be suitable for detection of other mutant BRAF variants within the activation segment in exon 15 in both melanoma and other tumors. To test this idea, we performed a literature search for all previously-published BRAF mutations in different human tumors using Pubmed (http://www.ncbi.nlm.nih.gov/pubmed). We found that the dispensation nucleotides T2A3C4 and C6 are required for detection of BRAF mutations affecting codon T599 [25], [33], [34], [36], [37], [40] (**Table 2**).

**Table 2:** Recognition patterns for 36 BRAF mutations by U-BRAF^V600^ assay.

	Mutation	Recognition Patterns						Unique properties of each mutationwithin one group	mt:wt ratio^1^	COSMIC database^2^
		C6	A10	C11	T12	A18	G19			
1	p.V600E(1)	–	–	–	–	–	–	A8 = Amt; T9 = Twt	A8^mt^ : T9^wt^	COSM476 [16]
	p.T599del	–	–	–	–	–	–	absence of A8; absence of mutant T2, C4 and A5	[I3–I5]^mt^ : A5^wt^	- [17]
	p.V600L	–	–	–	–	–	–	absence of A8; G7 = Gwt; T9 = [Twt +2Tmt]	[I13 - I7]^mt^ : G7^wt^	COSM33808 [18]
	p.V600M	–	–	–	–	–	–	absence of A8; G7 = Gwt; T9 = [Twt+Tmt]	[I13 - I7]^mt^ : G7^wt^	COSM1130 [19]
	p.V600R(2)	–	–	–	–	–	–	A5 = Awt; G13 = [Gwt +2Gmt]	A8^mt^ : T9^wt^	COSM1127 [20]
	p.K601E	–	–	–	–	–	–	absence of A8; G13 = [Gwt +2Gmt];A14 = [3Awt +2Amt]	[I13–I7]^mt^ :[2I7 - I13]^wt^	COSM478 [21]
	p.K601N	–	–	–	–	–	–	absence of A8; T9 = [Twt+Tmt]; A14 = [3Awt +2Amt]	[I15–I9]^mt^ :[2I9 - I15]^wt^	COSM1132 [22]
2	p.V600E;K601I	–	**+**	–	**+**	–	–	A8 : A10 = 3∶ 1	T12^mt^ : G13^wt^	COSM475 [23]
										COSM26491
	p.V600D	–	**+**	–	**+**	–	–	A8 : A10 = 1∶ 3	T12^mt^ : G13^wt^	COSM477 [16]
	p.V600G	–	**+**	–	**+**	–	–	G7 = Gwt +3Gmt; absence of A8; A10 = 3Amt; G13 = Gwt	T12^mt^ : G13^wt^	COSM6137 [19]
3	p.V600E(2)	–	–	**+**	**+**	–	**+**	A8 : T12 = 5∶ 1	T12^mt^ : G13^wt^	COSM475 [23]
	p.V600K	–	–	**+**	**+**	–	**+**	A5 = [Awt+3Amt]; A8 : T12 = 3∶ 1	T12^mt^ : G13^wt^	COSM473 [16]
	p.V600R(1)	–	–	**+**	**+**	–	**+**	A5 = [Awt +2Amt]; G7 = [Gwt +2Gmt];A8 : T12 = 3∶ 1	T12^mt^ : G13^wt^	COSM474 [16]
	p.V600_K601>E	–	–	**+**	**+**	–	**+**	A8 : T12 = 2∶ 1	T12^mt^ : G13^wt^	COSM1133 [24]
	p.TVKSR599_603>I	–	–	**+**	**+**	–	**+**	absence of mutant C4, A5, G7; absence of A8	C11^mt^ : G7^wt^	COSM30605 [25]
4	p.T599T;V600E	–	–	–	–	–	**+**	absence of mutant A5, G7; absence of A8	G19^mt^ : G7^wt^	COSM24963 [26]
										COSM476
	p.T599_V600>RE	–	–	–	–	–	**+**	absence of mutant C4, A5; A14 = [3Awt+Amt]	A8^mt^ : T9^wt^	- [27]
	p.K601R	–	–	–	–	–	**+**	absence of A8; A14 = [3Awt+Amt]	G19^mt^ : T15^wt^	COSM13625 [19]
	p.K601K	–	–	–	–	–	**+**	absence of A8; A14 = [3Awt +2Amt]	G19^mt^ : T15^wt^	COSM28507 [28]
5	p.K601Q	–	–	–	–	**+**	–	absence of A8; T9 = [Twt+Tmt]; A18 = 2Amt	½ A18^mt^ : T15^wt^	COSM1066665 [29]
	p.VKSRWS600_605>D	–	–	–	–	**+**	–	A8 = Amt; G13 = [Gwt +3Gmt]; C16 = [Cwt +3Cmt]	A18^mt^ : T17^wt^	COSM1129 [30]
	p.VKSRWS600_605>EK	–	–	–	–	**+**	–	A8 = 4Amt; G13 = [Gwt +4Gmt]; C16 = [Cwt +3Cmt]	A18^mt^ : T17^wt^	COSM306133 [31]
6	p.V600K;S602S	–	–	**+**	–	–	**+**	A8 = 3Amt	T17^mt^ : C11^wt^	COSM473 [26]
										COSM21611
	p.T599A	–	–	**+**	–	–	**+**	absence of A8	C11^mt^ : T9^wt^	- [32]
7	p.T599_V600insT(1)	–	**+**	**+**	–	–	**+**	absence of mutant G7; T9 = [Twt+Tmt]	C11^mt^ : C16^wt^	COSM30730 [33]
	p.T599_V600insV	–	**+**	**+**	–	–	**+**	absence of mutant A3, C4; T9 = [Twt +2Tmt]	A10^mt^ : A5^wt^	COSM21616 [34]
	p.V600>YM	–	**+**	**+**	–	–	**+**	absence of A8; absence of mutant G7, G13and T17	C11^mt^ : G13^wt^	- [35]
8	p.T599I;V600E	–	**+**	–	–	–	**+**	A14 = 3Awt+Amt; T17 = Twt	A10^mt^ : G7^wt^	COSM472 [36]
										COSM476
	p.A598_T599insKKGNFGLA	–	**+**	–	–	–	**+**	A3 = Awt +7Amt; absence of mutant A14	A10^mt^ : G7^wt^	- [27]
	p.T599_V600>IAL	–	**+**	–	–	–	**+**	absence of mutant A14; T17 = [Twt +3Tmt]	A10^mt^ : G7^wt^	COSM33780 [37]
9	p.T599_V600insTT	–	**+**	**+**	**+**	–	**+**	absence of A8; absence of mutant A5;G19 = 2Gmt	T12^mt^ : T15^wt^	COSM26459 [20]
	p.VKS600_602>DT	–	**+**	**+**	**+**	–	**+**	A8 = Amt; absence of mutant A14	T12^mt^ : T15^wt^	- [12]
10	p.T599_V600insT(2)	**+**	–	–	–	–	**+**	unique; C4 = A8	C11^mt^ : C16^wt^	COSM36245 [38]
11	p.T599_V600insDFLAGT	–	–	–	–	**+**	**+**	unique; T9 = [Twt +4Tmt]	A18^mt^ : ⅓C11^wt^	COSM26504 [24]
12	p.V600A	–	–	**+**	–	–	–	unique; absence of A8	C11^mt^ : T9^wt^	COSM18443 [19]
13	p.VKSRWS600_605>DV	–	–	–	–	–	**+**	G19 = 3Gmt; absence of mutant A14;T15 = [Twt +2Tmt]	A8^mt^ : T17^wt^	COSM33764 [39]

1 wt – wild type, mt – mutant; I – intensity value of correspondent nucleotide dispensation. A-peak reduction factor 0.9 should be taken into consideration.

2 Catalogue of Somatic Mutations in Cancer (COSMIC) database, version 62 (Wellcome Trust Sanger Institute).

Remarkably, the dispensation nucleotide C6, originally used as internal negative control, is thought to participate in the detection of p.T599_V600insT (c.A1797_1798insACA) [38] and, therefore, was added to the recognition patterns of U-BRAF^V600^ dispensation order (**Table 2**). Individual pyrograms were calculated for each mutation variant (**Table S3 in File S1**).

**Figure 4:**
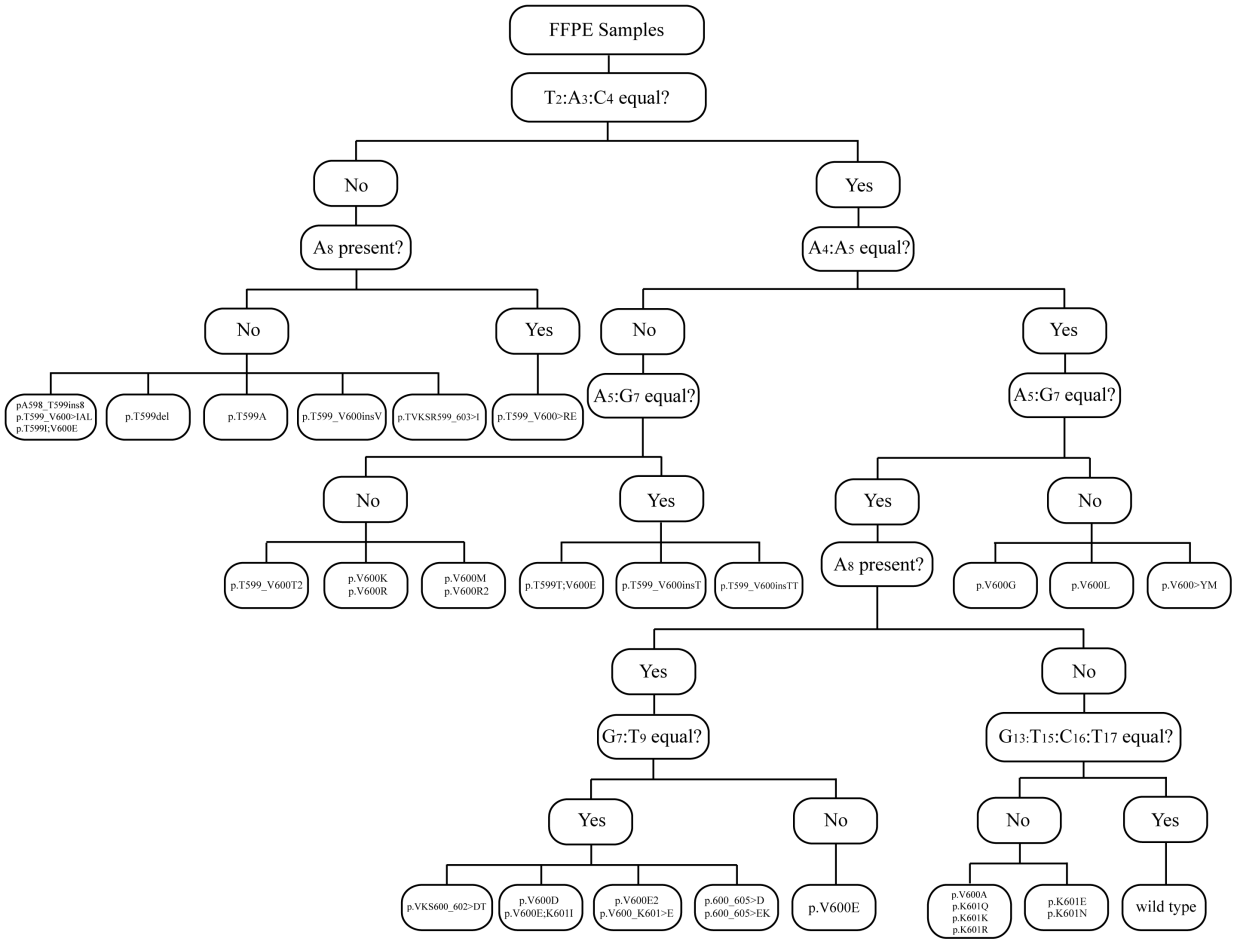
Algorithm for automated BRAF state classification of U-BRAF^V600^ pyrosequencing data analysis. Reduction factors for both A-peak and dispensation steps should be taken into consideration calculating individual peak intensities.

We demonstrate *in silico* that our dispensation order U-BRAF^V600^ is suitable for identification of other 31 previously-published BRAF mutation variants –36 variants in total including 5 mutations from the current study – affecting codons from T599 to S605 within the activation segment. According to recognition pattern signatures, we specified 9 groups as well as 4 unique mutation variants (**Table 2**). Importantly, each BRAF-mutated variant, including hypothetical one, consists of the features that are unique for each mutation within one group (**Table 2**), which enables U-BRAF^V600^ data analysis by the algorithm for BRAF state classification (**Figure 4** **)**.

In comparing our review of articles with the Catalogue of Somatic Mutations in Cancer (COSMIC) database [41], we identified several incorrect entries in the database, which represent either one mutation as two independent entries or one complex mutation as two different cases. Mutations p.T599T (COSM24963), p.T509I (COSM472), p.K601I (COSM26491) and p.S602S (COSM21611), which are described as individual mutations by COSMIC database, are in fact parts of complex mutations p.T599T;V600E [26], p.T599I;V600E [36], p.V600E;K601I [23], or p.V600E;S602S [26], respectively. Therefore, to distinguish a tandem mutation from other types of BRAF mutation, it might be necessary to annotate these particular BRAF mutants in the separate section as complex mutations within the COSMIC database.

Although the mutation p.K601del (COSM30594) is defined as a deletion of AAA-triplet at position 1801 to 1803 (c.1801_1803delAAA) [41], this mutation is in fact created by deletion of triplet TGA at position 1799 to 1801 (c.1799_1801delTGA), resulting in the complex mutation p.V600_K601>E (COSM1133) [24]. Furthermore, the mutation c.1794_1795insGTT [34] is represented as both p.A598_T599insV (COSM26625) and p.T599_V600insV (COSM21616).

Due to the absence of correspondent nucleotide sequences in the original publication, the unique mutations p.K601E;W604 and p.T599T;V600R published by Edlundh-Rose et al. [42] as well as p.V600DLAT published by Satoh et al. [32] were not included in the U-BRAF^V600^ analysis. Additionally, unpublished DNA sequencing data by Sadow et al. [43] made it impossible to annotate the misrepresented mutation “VKWRV600-604E” as p.V600_W604del (COSM37034) [41].

In summary, U-BRAF^V600^ approach takes advantage of gold standard Sanger sequencing to detect all mutation variants beyond V600E in a single assay, and according to our ultra-deep-sequencing validation, it is significantly more sensitive than Sanger sequencing. Moreover, mutations can be reliably distinguished from V600E mutation down to 2–3% mutant DNA in wild-type background (**Figure 3c**). In contrast to Sanger sequencing, the analysis of raw pyrosequencing data can be performed automatically using simple logical functions of a spreadsheet application (**Table S2 in File S1**). Furthermore, in **Figure 4** we present the algorithm for automation of BRAF state classification of U-BRAF^V600^ pyrosequencing data analysis taking into consideration the individual features of each mutation variant shown in **Table 2**. Thus, the single-reaction assay and data analysis automation makes U-BRAF^V600^ suitable for the assessment of large clinical sample sizes.

Taking all advantages together, we propose U-BRAF^V600^ approach as a universal diagnostic tool in the automated evaluation of metastatic melanoma and other tumors for their BRAF mutation state prior to targeted therapy.

## Supporting Information

Figure S1 **Comparison of BRAF mutation analyses.** (**a**) conventional pyrosequencing assay; (**b**) Sanger sequencing; **(c**) U-BRAF^V600^ pyrosequencing assay.
(EPS)

File S1 **Additional tables.** Table S1, Primer sequences and PCR conditions. Table S2, Spreadsheet for BRAF state detection by U-BRAF^V600^. Table S3, Pyrogram sequence patterns for 36 BRAF mutations detectable by U-BRAF^V600^ assay.
(PDF)

## References

[1] Balch CM, Gershenwald JE, Soong SJ, Thompson JF, Atkins MB, et al (2009) Final version of 2009 AJCC melanoma staging and classification. J Clin Oncol 27: 6199–6206. 19917835 10.1200/JCO.2009.23.4799 PMC2793035

[2] Lee JH, Choi JW, Kim YS (2011) Frequencies of BRAF and NRAS mutations are different in histological types and sites of origin of cutaneous melanoma: a meta-analysis. Br J Dermatol 164: 776–784. 21166657 10.1111/j.1365-2133.2010.10185.x

[3] Miller AJ, Mihm MC Jr (2006) Melanoma. N Engl J Med 355: 51–65. 16822996 10.1056/NEJMra052166

[4] Ellerhorst JA, Greene VR, Ekmekcioglu S, Warneke CL, Johnson MM, et al (2011) Clinical correlates of NRAS and BRAF mutations in primary human melanoma. Clin Cancer Res 17: 229–235. 20975100 10.1158/1078-0432.CCR-10-2276 PMC3022950

[5] Davies H, Bignell GR, Cox C, Stephens P, Edkins S, et al (2002) Mutations of the BRAF gene in human cancer. Nature 417: 949–954. 12068308 10.1038/nature00766

[6] Garnett MJ, Marais R (2004) Guilty as charged: B-RAF is a human oncogene. Cancer Cell 6: 313–319. 15488754 10.1016/j.ccr.2004.09.022

[7] Safaee AG, Jafarnejad SM, Tan L, Saeedi A, Li G (2012) The Prognostic Value of BRAF Mutation in Colorectal Cancer and Melanoma: A Systematic Review and Meta-Analysis. PLoS One 7: e47054. 23056577 10.1371/journal.pone.0047054 PMC3467229

[8] Chapman PB, Hauschild A, Robert C, Haanen JB, Ascierto P, et al (2011) Improved survival with vemurafenib in melanoma with BRAF V600E mutation. N Engl J Med 364: 2507–2516. 21639808 10.1056/NEJMoa1103782 PMC3549296

[9] Magoc T, Salzberg SL (2011) FLASH: fast length adjustment of short reads to improve genome assemblies. Bioinformatics 27: 2957–2963. 21903629 10.1093/bioinformatics/btr507 PMC3198573

[10] Jones DT, Jager N, Kool M, Zichner T, Hutter B, et al (2012) Dissecting the genomic complexity underlying medulloblastoma. Nature 488: 100–105. 22832583 10.1038/nature11284 PMC3662966

[11] Nakamura K, Oshima T, Morimoto T, Ikeda S, Yoshikawa H, et al (2011) Sequence-specific error profile of Illumina sequencers. Nucleic Acids Res 39: e90. 21576222 10.1093/nar/gkr344 PMC3141275

[12] Skorokhod A, Capper D, von Deimling A, Enk A, Helmbold P (2012) Detection of BRAF V600E mutations in skin metastases of malignant melanoma by monoclonal antibody VE1. J Am Acad Dermatol 67: 488–491. 22890732 10.1016/j.jaad.2012.03.022

[13] Kim SK, Kim DL, Han HS, Kim WS, Kim SJ, et al (2008) Pyrosequencing analysis for detection of a BRAFV600E mutation in an FNAB specimen of thyroid nodules. Diagn Mol Pathol 17: 118–125. 18382358 10.1097/PDM.0b013e31815d059d

[14] Heyries KA, Tropini C, Vaninsberghe M, Doolin C, Petriv OI, et al (2011) Megapixel digital PCR. Nat Methods 8: 649–651. 21725299 10.1038/nmeth.1640

[15] Chung KW, Yang SK, Lee GK, Kim EY, Kwon S, et al (2006) Detection of BRAFV600E mutation on fine needle aspiration specimens of thyroid nodule refines cyto-pathology diagnosis, especially in BRAF600E mutation-prevalent area. Clin Endocrinol (Oxf) 65: 660–666. 17054470 10.1111/j.1365-2265.2006.02646.x

[16] Spittle C, Ward MR, Nathanson KL, Gimotty PA, Rappaport E, et al (2007) Application of a BRAF pyrosequencing assay for mutation detection and copy number analysis in malignant melanoma. J Mol Diagn 9: 464–471. 17690212 10.2353/jmoldx.2007.060191 PMC1975103

[17] Schulten HJ, Salama S, Al-Mansouri Z, Alotibi R, Al-Ghamdi K, et al (2012) BRAF mutations in thyroid tumors from an ethnically diverse group. Hered Cancer Clin Pract 10: 10. 22925390 10.1186/1897-4287-10-10 PMC3434056

[18] Boulalas I, Zaravinos A, Delakas D, Spandidos DA (2009) Mutational analysis of the BRAF gene in transitional cell carcinoma of the bladder. Int J Biol Markers 24: 17–21. 19404918 10.1177/172460080902400103

[19] Lin J, Goto Y, Murata H, Sakaizawa K, Uchiyama A, et al (2011) Polyclonality of BRAF mutations in primary melanoma and the selection of mutant alleles during progression. Br J Cancer 104: 464–468. 21224857 10.1038/sj.bjc.6606072 PMC3049568

[20] Omholt K, Platz A, Kanter L, Ringborg U, Hansson J (2003) NRAS and BRAF mutations arise early during melanoma pathogenesis and are preserved throughout tumor progression. Clin Cancer Res 9: 6483–6488. 14695152

[21] Trovisco V, Soares P, Soares R, Magalhaes J, Sa-Couto P, et al (2005) A new BRAF gene mutation detected in a case of a solid variant of papillary thyroid carcinoma. Hum Pathol 36: 694–697. 16021577 10.1016/j.humpath.2005.04.011

[22] Helmke BM, Mollenhauer J, Herold-Mende C, Benner A, Thome M, et al (2004) BRAF mutations distinguish anorectal from cutaneous melanoma at the molecular level. Gastroenterology 127: 1815–1820. 15578519 10.1053/j.gastro.2004.08.051

[23] Indsto JO, Kumar S, Wang L, Crotty KA, Arbuckle SM, et al (2007) Low prevalence of RAS-RAF-activating mutations in Spitz melanocytic nevi compared with other melanocytic lesions. J Cutan Pathol 34: 448–455. 17518771 10.1111/j.1600-0560.2006.00646.x

[24] Hou P, Liu D, Xing M (2007) Functional characterization of the T1799–1801del and A1799–1816ins BRAF mutations in papillary thyroid cancer. Cell Cycle 6: 377–379. 17297294 10.4161/cc.6.3.3818

[25] Lupi C, Giannini R, Ugolini C, Proietti A, Berti P, et al (2007) Association of BRAF V600E mutation with poor clinicopathological outcomes in 500 consecutive cases of papillary thyroid carcinoma. J Clin Endocrinol Metab 92: 4085–4090. 17785355 10.1210/jc.2007-1179

[26] Deichmann M, Thome M, Benner A, Kirschner M, Hassanzadeh J, et al (2005) Preponderance of the oncogenic V599E and V599K mutations in B-raf kinase domain is enhanced in melanoma cutaneous/subcutaneous metastases. BMC Cancer 5: 58. 15935100 10.1186/1471-2407-5-58 PMC1164406

[27] Jung CK, Im SY, Kang YJ, Lee H, Jung ES, et al.. (2012) Mutational Patterns and Novel Mutations of the BRAF Gene in a Large Cohort of Korean Patients with Papillary Thyroid Carcinoma. Thyroid: 791–797. 10.1089/thy.2011.0123 22471241

[28] Deichmann M, Krahl D, Thome M, Wust K, Hassanzadeh J, et al (2006) The oncogenic B-raf V599E mutation occurs more frequently in melanomas at sun-protected body sites. Int J Oncol 29: 139–145. 16773193

[29] Sarkozy A, Carta C, Moretti S, Zampino G, Digilio MC, et al (2009) Germline BRAF mutations in Noonan, LEOPARD, and cardiofaciocutaneous syndromes: molecular diversity and associated phenotypic spectrum. Hum Mutat 30: 695–702. 19206169 10.1002/humu.20955 PMC4028130

[30] Cruz F III, Rubin BP, Wilson D, Town A, Schroeder A, et al (2003) Absence of BRAF and NRAS mutations in uveal melanoma. Cancer Res 63: 5761–5766. 14522897

[31] Hunter SM, Gorringe KL, Christie M, Rowley SM, Bowtell DD, et al (2012) Pre-invasive ovarian mucinous tumors are characterized by CDKN2A and RAS pathway aberrations. Clin Cancer Res 18: 5267–5277. 22891197 10.1158/1078-0432.CCR-12-1103

[32] Satoh T, Smith A, Sarde A, Lu HC, Mian S, et al (2012) B-RAF mutant alleles associated with Langerhans cell histiocytosis, a granulomatous pediatric disease. PLoS One 7: e33891. 22506009 10.1371/journal.pone.0033891 PMC3323620

[33] Eisenhardt AE, Olbrich H, Roring M, Janzarik W, Anh TN, et al (2010) Functional characterization of a BRAF insertion mutant associated with pilocytic astrocytoma. Int J Cancer 129(9): 2297–2303. 10.1002/ijc.25893 21190184

[34] Carta C, Moretti S, Passeri L, Barbi F, Avenia N, et al (2006) Genotyping of an Italian papillary thyroid carcinoma cohort revealed high prevalence of BRAF mutations, absence of RAS mutations and allowed the detection of a new mutation of BRAF oncoprotein (BRAF(V599lns)). Clin Endocrinol (Oxf) 64: 105–109. 16402937 10.1111/j.1365-2265.2005.02401.x

[35] Matsuse M, Mitsutake N, Tanimura S, Ogi T, Nishihara E, et al (2012) Functional characterization of the novel BRAF complex mutation, BRAF(V600delinsYM), identified in papillary thyroid carcinoma. Int J Cancer 132(3): 738–743. 22752848 10.1002/ijc.27709

[36] Gill M, Renwick N, Silvers DN, Celebi JT (2004) Lack of BRAF mutations in Spitz nevi. J Invest Dermatol 122: 1325–1326. 15140238 10.1111/j.0022-202X.2004.22530.x

[37] Chiosea S, Nikiforova M, Zuo H, Ogilvie J, Gandhi M, et al (2009) A novel complex BRAF mutation detected in a solid variant of papillary thyroid carcinoma. Endocr Pathol 20: 122–126. 19370421 10.1007/s12022-009-9073-3

[38] Schindler G, Capper D, Meyer J, Janzarik W, Omran H, et al (2011) Analysis of BRAF V600E mutation in 1,320 nervous system tumors reveals high mutation frequencies in pleomorphic xanthoastrocytoma, ganglioglioma and extra-cerebellar pilocytic astrocytoma. Acta Neuropathol 121: 397–405. 21274720 10.1007/s00401-011-0802-6

[39] Barzon L, Masi G, Boschin IM, Lavezzo E, Pacenti M, et al (2008) Characterization of a novel complex BRAF mutation in a follicular variant papillary thyroid carcinoma. Eur J Endocrinol 159: 77–80. 18426810 10.1530/EJE-08-0239

[40] Deichmann M, Thome M, Benner A, Naher H (2004) B-raf exon 15 mutations are common in primary melanoma resection specimens but not associated with clinical outcome. Oncology 66: 411–419. 15331929 10.1159/000079490

[41] Forbes SA, Bhamra G, Bamford S, Dawson E, Kok C, et al.. (2008) The Catalogue of Somatic Mutations in Cancer (COSMIC). Curr Protoc Hum Genet Chapter 10: Unit 11.1–26. 10.1002/0471142905.hg1011s57 PMC2705836 18428421

[42] Edlundh-Rose E, Egyhazi S, Omholt K, Mansson-Brahme E, Platz A, et al (2006) NRAS and BRAF mutations in melanoma tumours in relation to clinical characteristics: a study based on mutation screening by pyrosequencing. Melanoma Res 16: 471–478. 17119447 10.1097/01.cmr.0000232300.22032.86

[43] Sadow PM, Heinrich MC, Corless CL, Fletcher JA, Nose V (2010) Absence of BRAF, NRAS, KRAS, HRAS mutations, and RET/PTC gene rearrangements distinguishes dominant nodules in Hashimoto thyroiditis from papillary thyroid carcinomas. Endocr Pathol 21: 73–79. 20012784 10.1007/s12022-009-9101-3

