# Correction: Automated Universal BRAF State Detection within the Activation Segment in Skin Metastases by Pyrosequencing-Based Assay U-BRAF^V600^

**DOI:** 10.1371/annotation/b31c5248-91ad-4668-930a-24543b19d6e7

**Published:** 2013-06-19

**Authors:** Alexander Skorokhod, Peter Helmbold, Benedikt Brors, Peter Schirmacher, Alexander Enk, Roland Penzel

The Titles and Legends for Figures 2 and 3 were incorrectly switched. The title and legend for Figure 2 should appear with Figure 3, and the title and legend for Figure 3 should appear with Figure 2. The figures themselves are in correct order. 

