# Supplementary material for: Automated Universal BRAF State Detection within the Activation Segment in Skin Metastases by Pyrosequencing-Based Assay U-BRAFV600
Source: PLoS One. 2013 Mar 26;8(3):e59221. doi: 10.1371/journal.pone.0059221 (PMC3608589; doi:10.1371/journal.pone.0059221)
Supplement: File S1 — Additional tables. Table S1, Primer sequences and PCR conditions. Table S2, Spreadsheet for BRAF state detection by U-BRAFV600. Table S3, Pyrogram sequence patterns for 36 BRAF mutations detectable by U-BRAFV600 assay. (PDF) [file pone.0059221.s002.pdf]

Table S1. Primer sequences and PCR conditions

Skorokhod et al.

Primer	Sequence	PCR conditions
U-BRAF-F	5'-ATGCTTGCTCTGATAGGAATG-3'	98°C – 2 min<br>98°C – 10 sec<br>56°C – 30 sec<br>72°C – 20 sec<br>35 cycles
U-BRAF-Pyro-R	Biotin-TEG-5'-AGCATCTCAGGGCCAAAAAT-3'	
U-BRAF-599-Seq	5'-AATAGGTGATTTTGGTCTAGC-3'	
U-BRAF-600-Seq	5'-GGTGATTTTGGTCTAGCTAC-3'	
BRAF-15F-Seq	5'-TGCTTGCTCTGATAGGAAAATG-3'	95°C – 20 sec<br>55°C – 15 sec<br>60°C – 1 min<br>25 cycles
BRAF-15R-Seq	5'-AGCATCTCAGGGCCAAAAAT-3'	
MiSeq-Rev	5'- TGGAGTTCAGACGTGTGCTCTTCCGATCTtagcctcaattcttacCATCCAC-3'	98°C - 2 min<br>98°C - 10 sec<br>56°C - 30 sec<br>72°C - 20 sec<br>25 cycles
MiSeq-F01	5'-TTTCCCTACACGACGCTCTTCCGATCTGATCtttcctttacttactacacctcag-3'	
MiSeq-F02	5'-TTTCCCTACACGACGCTCTTCCGATCTGACAtttcctttacttactacacctcag-3'	
MiSeq-F03	5'-TTTCCCTACACGACGCTCTTCCGATCTGTCCtttcctttacttactacacctcag-3'	
MiSeq-F04	5'-TTTCCCTACACGACGCTCTTCCGATCTGCTAtttcctttacttactacacctcag-3'	
MiSeq-F05	5'-TTTCCCTACACGACGCTCTTCCGATCTCCGCtttcctttacttactacacctcag-3'	
MiSeq-F06	5'-TTTCCCTACACGACGCTCTTCCGATCTCAGAtttcctttacttactacacctcag-3'	
MiSeq-F07	5'-TTTCCCTACACGACGCTCTTCCGATCTCGAGtttcctttacttactacacctcag-3'	
MiSeq-F08	5'-TTTCCCTACACGACGCTCTTCCGATCTCATAtttcctttacttactacacctcag-3'	
MiSeq-F09	5'-TTTCCCTACACGACGCTCTTCCGATCTTGTAtttcctttacttactacacctcag-3'	
MiSeq-F10	5'-TTTCCCTACACGACGCTCTTCCGATCTTCCTtttcctttacttactacacctcag-3'	
MiSeq-F11	5'-TTTCCCTACACGACGCTCTTCCGATCTTCTTtttcctttacttactacacctcag-3'	
MiSeq-F12	5'-TTTCCCTACACGACGCTCTTCCGATCTTAGTtttcctttacttactacacctcag-3'	
MiSeq-F13	5'-TTTCCCTACACGACGCTCTTCCGATCTATCTtttcctttacttactacacctcag-3'	
MiSeq-F14	5'-TTTCCCTACACGACGCTCTTCCGATCTACACtttcctttacttactacacctcag-3'	
MiSeq-F15	5'-TTTCCCTACACGACGCTCTTCCGATCTACGAtttcctttacttactacacctcag-3'	
MiSeq-F16	5'-TTTCCCTACACGACGCTCTTCCGATCTAGAGTtttcctttacttactacacctcag-3'	
MiSeq-F17	5'-TTTCCCTACACGACGCTCTTCCGATCTGTAAGtttcctttacttactacacctcag-3'	
MiSeq-F18	5'-TTTCCCTACACGACGCTCTTCCGATCTACTGCtttcctttacttactacacctcag-3'	
MiSeq-F19	5'-TTTCCCTACACGACGCTCTTCCGATCTAAGGAtttcctttacttactacacctcag-3'	

MiSeq-F20	5‘-TTTCCCTACACGACGCTCTTCCGATCTCTAAGtttcctttacttactacacctcag-3‘	
MiSeq-F21	5‘-TTTCCCTACACGACGCTCTTCCGATCTTGAACtttcctttacttactacacctcag-3‘	
MiSeq-F22	5‘-TTTCCCTACACGACGCTCTTCCGATCTTGCTAtttcctttacttactacacctcag-3‘	
MiSeq-F23	5‘-TTTCCCTACACGACGCTCTTCCGATCTTGTTCtttcctttacttactacacctcag-3‘	
MiSeq-F24	5‘-TTTCCCTACACGACGCTCTTCCGATCTTAAGAtttcctttacttactacacctcag-3‘	
MiSeq-F25	5‘-TTTCCCTACACGACGCTCTTCCGATCTCTAATtttcctttacttactacacctcag-3‘	
MiSeq-F26	5‘-TTTCCCTACACGACGCTCTTCCGATCTGAGTGtttcctttacttactacacctcag-3‘	
MiSeq-F27	5‘-TTTCCCTACACGACGCTCTTCCGATCTACTGAtttcctttacttactacacctcag-3‘	
MiSeq-F28	5‘-TTTCCCTACACGACGCTCTTCCGATCTATTCCtttcctttacttactacacctcag-3‘	
MiSeq-F29	5‘-TTTCCCTACACGACGCTCTTCCGATCTACATCtttcctttacttactacacctcag-3‘	
MiSeq-F30	5‘-TTTCCCTACACGACGCTCTTCCGATCTGCCTAtttcctttacttactacacctcag-3‘	
MiSeq-F31	5‘-TTTCCCTACACGACGCTCTTCCGATCTCGATGTtttcctttacttactacacctcag-3‘	
MiSeq-F32	5‘-TTTCCCTACACGACGCTCTTCCGATCTTTAGGCtttcctttacttactacacctcag-3‘	
MiSeq-F33	5‘-TTTCCCTACACGACGCTCTTCCGATCTTGACCAtttcctttacttactacacctcag-3‘	
MiSeq-F34	5‘-TTTCCCTACACGACGCTCTTCCGATCTGCCAATtttcctttacttactacacctcag-3‘	
MiSeq-F35	5‘-TTTCCCTACACGACGCTCTTCCGATCTACTTGAtttcctttacttactacacctcag-3‘	
MiSeq-F36	5‘-TTTCCCTACACGACGCTCTTCCGATCTGTTCAGtttcctttacttactacacctcag-3‘	
MiSeq-F37	5‘-TTTCCCTACACGACGCTCTTCCGATCTTAGCTTtttcctttacttactacacctcag-3‘	
MiSeq-F38	5‘-TTTCCCTACACGACGCTCTTCCGATCTCTTGTAtttcctttacttactacacctcag-3‘	
MiSeq-F39	5‘-TTTCCCTACACGACGCTCTTCCGATCTAGTCAAtttcctttacttactacacctcag-3‘	
MiSeq-F40	5‘-TTTCCCTACACGACGCTCTTCCGATCTAGTTCCtttcctttacttactacacctcag-3‘	
MiSeq-F41	5‘-TTTCCCTACACGACGCTCTTCCGATCTATGTCAtttcctttacttactacacctcag-3‘	
MiSeq-F42	5‘-TTTCCCTACACGACGCTCTTCCGATCTCCGTCCtttcctttacttactacacctcag-3‘	
MiSeq-F43	5‘-TTTCCCTACACGACGCTCTTCCGATCTGTCCGCtttcctttacttactacacctcag-3‘	
MiSeq-F44	5‘-TTTCCCTACACGACGCTCTTCCGATCTGTGAAAtttcctttacttactacacctcag-3‘	
MiSeq-F45	5‘-TTTCCCTACACGACGCTCTTCCGATCTGTTTCGtttcctttacttactacacctcag-3‘	
MiSeq-F46	5‘-TTTCCCTACACGACGCTCTTCCGATCTTAAGGCGtttcctttacttactacacctcag-3‘	
MiSeq-F47	5‘-TTTCCCTACACGACGCTCTTCCGATCTCGTACTAtttcctttacttactacacctcag-3‘	
MiSeq-F48	5‘-TTTCCCTACACGACGCTCTTCCGATCTAGGCAGAtttcctttacttactacacctcag-3‘	
MiSeq-F49	5‘-TTTCCCTACACGACGCTCTTCCGATCTTCCTGAGtttcctttacttactacacctcag-3‘	

MiSeq-F50	5‘-TTTCCCTACACGACGCTCTTCCGATCTGGACTCCtttcctttacttactacacctcag-3‘	
MiSeq-F51	5‘-TTTCCCTACACGACGCTCTTCCGATCTTAGGCATtttcctttacttactacacctcag-3‘	
MiSeq-F52	5‘-TTTCCCTACACGACGCTCTTCCGATCTCTCTCTAtttcctttacttactacacctcag-3‘	
MiSeq-F53	5‘-TTTCCCTACACGACGCTCTTCCGATCTCAGAGAGtttcctttacttactacacctcag-3‘	
MiSeq-F54	5‘-TTTCCCTACACGACGCTCTTCCGATCTGCTACGCtttcctttacttactacacctcag-3‘	
MiSeq-F55	5‘-TTTCCCTACACGACGCTCTTCCGATCTCGAGGCTtttcctttacttactacacctcag-3‘	
MiSeq-F56	5‘-TTTCCCTACACGACGCTCTTCCGATCTAAGAGGCtttcctttacttactacacctcag-3‘	
MiSeq-F57	5‘-TTTCCCTACACGACGCTCTTCCGATCTGTAGAGGtttcctttacttactacacctcag-3‘	
MiSeq-F58	5‘-TTTCCCTACACGACGCTCTTCCGATCTTAGATCGtttcctttacttactacacctcag-3‘	
MiSeq-F59	5‘-TTTCCCTACACGACGCTCTTCCGATCTCTGACTTtttcctttacttactacacctcag-3‘	
MiSeq-F60	5‘-TTTCCCTACACGACGCTCTTCCGATCTTATCCTCtttcctttacttactacacctcag-3‘	
MiSeq-F61	5‘-TTTCCCTACACGACGCTCTTCCGATCTATCACGACtttcctttacttactacacctcag-3‘	
MiSeq-F62	5‘-TTTCCCTACACGACGCTCTTCCGATCTACAGTGGTtttcctttacttactacacctcag-3‘	
MiSeq-F63	5‘-TTTCCCTACACGACGCTCTTCCGATCTCAGATCCAtttcctttacttactacacctcag-3‘	
MiSeq-F64	5‘-TTTCCCTACACGACGCTCTTCCGATCTACAAACGGtttcctttacttactacacctcag-3‘	
MiSeq-F65	5‘-TTTCCCTACACGACGCTCTTCCGATCTACCCAGCAtttcctttacttactacacctcag-3‘	
MiSeq-F66	5‘-TTTCCCTACACGACGCTCTTCCGATCTAACCCCTCtttcctttacttactacacctcag-3‘	
MiSeq-F67	5‘-TTTCCCTACACGACGCTCTTCCGATCTCCCAACCTtttcctttacttactacacctcag-3‘	
MiSeq-F68	5‘-TTTCCCTACACGACGCTCTTCCGATCTCACCACACtttcctttacttactacacctcag-3‘	
MiSeq-F69	5‘-TTTCCCTACACGACGCTCTTCCGATCTGAAACCCAtttcctttacttactacacctcag-3‘	
MiSeq-F70	5‘-TTTCCCTACACGACGCTCTTCCGATCTTGTGACCAtttcctttacttactacacctcag-3‘	
MiSeq-F71	5‘-TTTCCCTACACGACGCTCTTCCGATCTAGGGTCAAtttcctttacttactacacctcag-3‘	
MiSeq-F72	5‘-TTTCCCTACACGACGCTCTTCCGATCTAGGAGTGGtttcctttacttactacacctcag-3‘	
MiSeq-F73	5‘-TTTCCCTACACGACGCTCTTCCGATCTCTAGAACAtttcctttacttactacacctcag-3‘	
MiSeq-F74	5‘-TTTCCCTACACGACGCTCTTCCGATCTTAAGTTCCtttcctttacttactacacctcag-3‘	
MiSeq-F75	5‘-TTTCCCTACACGACGCTCTTCCGATCTTAGACCTAtttcctttacttactacacctcag-3‘	
Universal Adapter	5’-AATGATACGGCGACCACCGAGATCTACACTCTTTCCCTACACGACGCTCTTCCGATCT-3’	98°C – 1 min<br>98°C – 10 sec<br>54°C – 30 sec<br>72°C – 20 sec<br>25 cycles
TruSeq Adapter	5`-CAAGCAGAAGACGGCATACGAGATCGTGACTGGAGTTCAGACGTGTGCTCTTCCGATCT-3’	

Table S2. Spreadsheet for BRAF state detection by U-BRAF^{V600} Skorokhod et al.

No.	Sample	Mutation	mt:wt ratio*	Recognition patterns**						Dispensation order																		
				C_6	A_{10}	C_{11}	T_{12}	A_{18}	G_{19}	G_1	T_2	A_3	C_4	A_6	C_6	G_7	A_8	T_9	A_{10}	C_{11}	T_{12}	G_{13}	A_{14}	T_{15}	C_{16}	T_{17}	A_{18}	G_{19}
1	**1A**	V600E	24	-	-	-	-	-	-	1.18	95.68	104.91	93.86	105.89	0.59	94.56	27.01	75.8	3.58	4.56	4.3	86.42	257.58	81.15	84.37	78.61	2.61	6.37
2	**1B**	V600E	25	-	-	-	-	-	-	1.43	62.59	69.77	63.17	69.32	0.64	60.34	18.88	49.89	2.24	3.38	3.18	55.97	169.19	54.29	55.38	53.43	1.8	4.72
3	**2A**	V600E	25	-	-	-	-	-	-	1.06	66.45	75.81	64.62	77.09	0.62	64.86	19.42	51.53	2.45	3.71	3.38	59.64	182.55	55.05	59.27	53.11	2.14	5.09
4	**2B**	V600E	26	-	-	-	-	-	-	1.36	44.31	50	46.44	49.94	0.62	44.99	13.59	34.52	1.93	1.78	1.83	41.15	123	39.96	39.81	37.85	1.33	3.33
5	**3A**	V600E	16	-	-	-	-	-	-	0.77	36.03	43.36	36.55	43.07	0.43	37.34	7.07	34.14	3.11	3.3	3.61	32.86	98.74	30.38	33.58	28.85	1.43	4.07
6	**3B**	V600E	20	-	-	-	-	-	-	1.11	58.07	67.28	61.69	67.25	0.68	59.82	14.75	52	2.08	3.3	3.3	54.29	165.1	52.92	53.49	50.97	2.07	4.63
7	**3C**	V600E	20	-	-	-	-	-	-	1.18	33.87	40.13	32.82	38.67	0.33	33.34	8.39	29.93	1.84	2.62	2.54	30.86	92.06	28.92	30.33	28.93	1.48	2.89
8	**4A**	V600E	53	-	-	-	-	-	-	1.23	59.18	66.44	60.73	65.28	0.59	58.94	35.26	27.95	2.01	1.68	2.62	55.23	166.54	51.34	54.24	51.65	2.06	3.34
9	**4B**	V600E	59	-	-	-	-	-	-	1.16	67.33	72.84	65.28	71.19	0.5	65.69	43.66	27.82	3.23	1.86	5.09	60.94	182.55	57.89	61.78	58.49	3.23	3.48
10	**5A**	WT	6	-	-	-	-	-	-	0.88	53.09	61.75	52.05	58.93	0.42	51.67	3.55	52.14	1.96	1.8	1.87	49.87	153.09	46.01	48.52	45.96	1.62	2.99
11	**6A**	WT	4	-	-	-	-	-	-	1.31	87.85	95.54	84.35	93.5	0.81	80.64	3.37	80.79	2.75	2.44	2.09	79.84	241.07	74.25	77.09	73.92	2	4.08
12	**6B**	WT	4	-	-	-	-	-	-	0.96	70.72	81.74	69.65	77.94	0.52	68.61	3	69.32	2.98	1.85	1.83	65.87	204.39	62.81	64.17	61.88	1.84	3.46
13	**6C**	WT	3	-	-	-	-	-	-	1.24	96.69	105.96	93.5	103.14	0.74	88.4	3.3	88.39	2.8	2.38	2.31	87.16	266.26	84.27	84.19	82.59	2.21	4.7
14	**7A**	V600E	33	-	-	-	-	-	-	1.05	79.42	90.85	81.69	89.03	0.86	80.54	30.55	55.13	3.07	2.79	2.48	74.09	225.83	71.53	71.35	70.27	2.44	4.27
15	**8A**	V600E	37	-	-	-	-	-	-	1.14	52.3	59.58	51.33	57.45	0.5	49.31	21.95	33.44	1.55	1.98	1.45	47.42	145.47	46.24	45.25	45.25	1.63	3.13
16	**8B**	V600E	18	-	-	-	-	-	-	1.02	63.35	72.8	64.62	72.57	0.43	64.96	13.14	55.07	2.39	2.02	1.84	60.47	181.43	57.47	58.8	54.6	1.7	3.43
17	**8C**	V600E	26	-	-	-	-	-	-	0.86	18.86	23.93	18.48	22.41	0.45	20.09	5.99	15.08	0.77	0.67	0.77	18.75	56.17	16.79	17.09	15.7	1.3	1.19
18	**8D**	V600E	25	-	-	-	-	-	-	1.12	75.32	83.77	79.09	83.19	0.77	74.99	21.04	57.92	2.57	1.71	1.18	70.95	213.04	69.84	67.46	68.01	2	3.82
19	**8E**	V600E	35	-	-	-	-	-	-	1.05	75.47	87.22	75.75	84.67	0.53	76.19	30.29	50.12	2.69	2.41	2.34	70.64	217.04	67.02	68.46	63.43	2.47	3.72
20	**9A**	WT	7	-	-	-	-	-	-	1.07	64.5	73.85	65.07	70.63	0.61	62.79	5.04	63.64	2.33	2.53	2.59	58.13	180.96	58.64	57.52	57.24	1.9	3.64
21	**9B**	WT	5	-	-	-	-	-	-	0.91	63.03	72.65	60.9	70.9	0.55	61.35	3.62	59.09	1.81	1.86	2.2	59.73	182.38	54.06	57.9	54.08	1.9	2.81
22	**10A**	V600E	56	-	-	-	-	-	-	1.27	73.42	80.53	75.94	82.34	0.69	73.21	47.67	33.93	1.91	2.91	2.5	67.98	204.66	65.2	67.03	61.81	1.97	4.61
23	**10B**	V600E	62	-	-	-	-	-	-	0.89	48.63	57.07	48.25	55.64	0.39	48.86	34.7	18.82	1.6	1.67	1.86	46.44	140.13	41.29	44.75	41.5	2.43	2.55
24	**10C**	V600E	45	-	-	-	-	-	-	1.02	48.39	56.05	51.66	57.52	0.54	50.06	28.01	30.39	1.38	2.95	2.7	45.16	137.81	44.19	45.93	41.63	1.56	3.92
25	**11A**	WT	5	-	-	-	-	-	-	1.62	91.69	100.09	93.12	96.52	0.91	90.98	4.98	90.08	3.53	3.49	5.19	83.92	245.99	78.92	79.82	76.87	2.45	7.18
26	**11B**	WT	4	-	-	-	-	-	-	2.11	109.8	119.26	108.1	114.65	0.87	105.9	4.49	101.68	3.47	2.76	1.78	99.44	292.79	92.73	93.38	89.8	2.45	6.89
27	**11C**	WT	5	-	-	-	-	-	-	1.61	98.55	108.76	99.96	105.36	0.93	97.98	4.96	94.21	3.88	2.76	2.04	91.96	271.47	85.85	86.18	81.45	2.58	6.7
28	**12A**	WT	3	-	-	-	-	-	-	1.08	102.91	113.56	101.33	108.21	0.48	98.53	3.07	98.67	4.27	3.38	2.95	92.46	279.45	87.92	88.1	86.92	2.47	5.68
29	**13A**	WT	4	-	-	-	-	-	-	1.51	103.73	113.56	105.54	111.96	0.88	103.11	4.15	100.83	3.84	3.32	3.18	96.48	286.52	90.12	90.53	87.4	2.61	6.14
30	**13B**	WT	4	-	-	-	-	-	-	1.55	85.08	93.08	83.2	88.72	0.62	81.74	3.43	80.01	3.43	2.24	2.13	75.29	227.92	71.72	71.95	69.89	1.95	5.22
31	**13C**	WT	4	-	-	-	-	-	-	1.76	95.84	104.99	96.31	101.84	0.93	94.96	3.93	90.01	3.31	2.31	1.67	89.14	262.08	83.36	83.73	80.66	2.31	5.73
32	**13D**	WT	4	-	-	-	-	-	-	1.26	101.25	111.38	102.29	106.85	0.6	99.62	3.89	96.34	2.53	1.82	2.03	92.83	276.19	87.16	88.35	84.38	1.78	6.16
33	**14A**	VKS>DT	33	-	+	+	+	-	+	1.47	111.28	118.5	110.63	115.04	0.81	108.02	40.21	103.59	37.51	35.38	34.48	68.63	199.43	65.01	94.25	60.84	2.54	36.72
34	**14B**	VKS>DT	23	-	+	+	+	-	+	2.1	106.11	113.95	105.36	111.28	1.01	102.98	26.1	99.29	25.51	24.91	23.19	76.04	221.82	70.35	89.18	67.14	3.52	27.24
35	**14C**	VKS>DT	37	-	+	+	+	-	+	1.51	98.69	109.28	100.56	104.59	0.77	97.3	40.49	95.25	39.57	36.39	35.29	58.82	170.12	56.12	86.18	52.64	3.13	36.96
36	**14D**	VKS>DT	24	-	+	+	+	-	+	2.14	90.45	97.55	87.82	95.19	0.8	87.26	22.49	84.62	21.16	21	19.82	64.45	190.13	59.5	75.98	58.18	2.43	21.27
37	**14E**	VKS>DT	35	-	+	+	+	-	+	1.49	91.2	100.37	91.72	96.77	0.83	90.32	36.97	84.26	32.78	32.15	30.8	56.14	161.59	51.59	78.79	49.28	2.47	33.78
38	**15A**	V600E	9	-	-	-	-	-	-	1.53	82.14	88.22	79.16	85.1	1.03	78.37	8.16	76.72	3.75	2.25	4.04	73.05	211.77	68.23	68.98	66.41	2.48	6.24
39	**15B**	WT	5	-	-	-	-	-	-	1.25	89.48	99.32	88.68	95.29	0.65	88.13	4.4	83.49	2.22	3.07	1.53	81.28	243.06	77.61	78.71	76.04	2.59	6.86
40	**16A**	V600E	21	-	-	-	-	-	+	1.42	89.17	97.88	87.72	103.4	1.03	87.22	23.82	82.24	2.48	7.87	8.47	76.38	224.49	72.2	77	70.02	1.89	10.76
41	**16B**	V600E	24	-	-	-	-	-	+	1.73	106.07	116.58	107.61	123.83	1.08	106.01	32.92	92.49	4.08	8.39	7.63	93.68	273.22	88.57	92.28	83	3.09	12.44
42	**17A**	V600K	10	-	-	+	+	-	+	1.37	92.6	102.03	92.09	105.26	0.7	91.86	18.88	87.14	3.83	9.19	8.97	80.47	239.82	74.57	80.53	75.2	3.14	9.55
43	**17B**	V600K	17	-	-	+	+	-	+	2.22	98.8	108.33	99.21	132.65	0.97	96.65	45.9	93.52	3.8	16.1	16.29	77.78	231.88	73.63	85.46	72.63	2.74	17.22

44	**18A**	V600E	28	-	-	-	-	-	-	2.1	87.8	96.81	85.66	93.33	0.66	86.46	27.95	65.87	3.56	4.67	4.69	79.04	238.53	73.07	76.32	73.79	2.72	5.83
45	**19A**	V600E	11	-	-	-	-	-	-	1.96	63.29	71.27	64.49	71.35	0.76	64.66	8.6	62	2.84	4.16	4.31	58.2	176.46	54.86	56.34	52.58	2.01	4.96
46	**20A**	V600E	17	-	-	-	-	-	-	1.32	82.49	92.35	81.51	93.16	0.7	81.01	17.14	76.61	4.97	6.35	5.29	71.28	215.61	69.5	71.78	68.28	2.76	7.92
47	**20B**	V600E	13	-	-	-	-	-	-	1.85	64.59	72.81	67.57	72.46	0.93	66.99	10.39	62.79	4.8	3.91	5.02	59.92	178.05	57.47	59.66	55.93	2.47	5.51
48	**20C**	V600E	13	-	-	-	-	-	-	1.21	84.24	92.87	82.61	91.32	0.61	82.53	12.47	77.49	3.13	4.47	3.81	73.88	221.5	70.59	72.83	68.7	1.69	7.33
49	**20D**	V600E	22	-	-	-	-	-	+	1.68	77.22	83.08	77.12	82.48	0.86	76.76	20.42	66.57	4.34	4.33	5.33	68.36	194.83	61.54	66.71	60.56	1.73	9.62
50	**20E**	V600E	11	-	-	-	-	-	-	3.58	78.41	88.49	80.76	87.02	0.81	78.92	10.18	75.59	3.25	5.01	6.14	72.36	211.39	67.06	70.41	66.62	2.36	6.03
51	**21A**	V600E2	27	-	-	+	+	-	+	1.75	81.03	89.5	78.42	86.04	0.95	79.47	99.69	74.31	3.77	20.91	20.55	54.95	161.01	53.37	70.18	51.29	2.4	21.4
52	**21B**	V600E2	34	-	-	+	+	-	+	1.32	73.55	81.17	74.4	78.06	0.48	73.22	112.83	67.97	3.89	24.45	24.2	46.45	135.42	44.93	65.78	45.13	2.98	23.45
53	**22A**	V600K	39	-	-	+	+	-	+	1.67	77.91	86.48	76.2	142.98	0.91	76.31	97.37	70.04	2.31	29.69	28.8	45.34	125.11	41.57	66.77	39.63	1.84	31.67
54	**22B**	V600K	39	-	-	+	+	-	+	2.71	86.2	95.07	87.51	159.07	0.73	84.74	105.78	78.76	3.69	34.41	32.45	49.91	139.58	44.33	75.41	45.63	1.52	34.74
55	**23A**	WT	4	-	-	-	-	-	-	2.21	76.55	84.14	76.82	80.13	0.79	77.81	3.75	72.15	1.66	1.85	3.21	72.27	207.59	65.25	68.02	65.68	1.58	4.77
56	**24A**	V600E	14	-	-	-	-	-	+	1.45	72	82.51	72.47	84.92	0.78	72.7	18.31	66.89	2.71	5.58	5.45	65.87	185.41	58.93	64.24	60.03	1.42	9.21
57	**24B**	V600E	9	-	-	-	-	-	-	2.87	87.17	96.54	84.36	92.14	0.7	85.79	9.18	80.02	2.5	2.79	2.83	80.35	228.16	76.47	76.25	74.35	2.58	5.97
58	**25A**	V600E	16	-	-	-	-	-	-	2.14	82.83	91.79	83.26	87.55	0.91	82.71	15.67	73.08	5.13	5.87	4.71	75.33	222.24	72.91	74.14	70.28	2.84	6.96
59	**26A**	WT	6	-	-	-	-	-	-	1.69	78.67	87.18	77.33	82.82	0.79	76.52	5.1	75.43	2.96	3.65	6.91	72.03	213.36	66.97	69.84	73.89	2.77	5
60	**26B**	WT	5	-	-	-	-	-	-	1.84	89.76	99.89	92.27	96.51	1.1	91.32	4.7	88.14	3.01	3.46	2.53	85.46	249.56	79.06	80.78	75.78	1.99	6.05
61	**26C**	WT	5	-	-	-	-	-	-	1.63	85.56	98.17	85.11	90.64	0.86	84.2	4.54	84.19	3.18	4.72	2.05	78.78	238.44	74.05	75.5	71.55	1.94	7.19
62	**26D**	WT	4	-	-	-	-	-	-	1.84	83.17	93.96	86.08	91.69	0.88	85.95	4.16	81.87	2.76	2.4	1.79	81.87	234.83	76.72	76.62	73.4	2.8	5.63
63	**26E**	WT	7	-	-	-	-	-	-	1.62	90.14	100.59	91.39	96.52	0.69	92.13	6.94	88.38	2.27	6.92	2.08	83.5	248.5	84.34	83.24	80.34	3.85	6.7
64	**26F**	WT	5	-	-	-	-	-	-	1.68	74.78	84.64	77.31	85.68	2.41	78.97	3.98	75.12	2.72	4.99	6.19	70.99	208.99	65.15	68.36	65.85	3.07	5.35
65	**26G**	WT	6	-	-	-	-	-	-	1.75	100.66	110.19	102.55	107.48	1	99.78	7.24	93.79	3.27	4.03	3.03	92.45	272.69	88.9	90.17	87.19	3.29	5.94
66	**26H**	WT	5	-		-	-	-	-	1.66	93.59	106.96	100.03	101.82	0.92	94.05	5.24	90.8	2.78	3.01	5.12	88.28	262.03	81.49	84.62	81.82	2.66	4.84
67	**27A**	V600K	49	-	-	+	+	-	+	1.67	80.57	87.73	85.82	159.57	6.87	78.66	107.48	83.99	2.47	39.9	41.28	42.76	124.47	38.92	71.81	42.61	4.4	34.23
68	**27B**	V600K	43	-	-	+	+	-	+	1.59	81.85	91.54	86.26	157.76	4.69	81.39	106.03	76.39	2.3	35.16	34.51	46.43	135.39	43.01	75.13	47.24	2.67	33.52
69	**27C**	V600K	47	-	-	+	+	-	+	3.78	72.63	88.07	77.28	140.87	0.84	69.72	95.18	72.93	2.13	33.87	35.19	38.91	117.47	37.26	65.05	39.77	3.7	28.97
70	**27D**	V600K	42	-	-	+	+	-	+	2.11	80.83	87.83	78.59	150.29	0.71	79.15	104.51	73.67	2	33.88	32.2	44.45	124.71	40.79	72.49	39.23	3.05	33.77
71	**27E**	V600K	56	-	-	+	+	-	+	1.58	80.61	91.03	78.99	174.31	0.81	84.13	137.25	75.52	2.32	44.87	41.95	33.32	100.26	38.37	73.63	35.77	3.86	42.21
72	**28A**	V600E	21	-	-	-	-	-	+	1.53	69.73	79.4	70.89	82.18	0.71	74.21	19.3	64.31	1.91	5.51	5.04	65	181.3	59.42	62.26	56.32	1.6	12.35
73	**28B**	V600E	26	-	-	-	-	-	-	1.33	41.4	47.58	40.42	48.51	1.25	39.47	14.67	37.4	3.16	4.29	3.28	36.74	109.94	37.75	36.83	36.4	4.08	4.28
74	**29A**	V600E;K601I	61	-	+	-	+	-	-	1.41	72.23	93.06	75.05	82.17	0.95	76.36	134.48	71.39	45.65	7.47	48.49	31.53	97.31	30.5	72.37	74.01	4.48	3.12
75	**29B**	V600E;K601I	39	-	+	-	+	-	-	1.7	103.99	116.08	101.72	112.57	1.72	99.88	118.34	95.47	40.88	4.96	38.2	60.55	182.87	63.42	89.91	93.12	3.55	5.02
76	**A549**	WT	7	-	-	-	-	-	-	1.08	36.92	43.43	34.35	40.7	0.75	34.78	2.89	34.63	0.83	1.15	1.14	33.68	105.32	34.07	31.76	32.78	0.79	1.45
77	**HeLa**	WT	6	-	-	-	-	-	-	3.01	88.58	103.43	93.2	99.68	0.91	91.14	6.47	88.41	3.55	3.54	3.04	86.97	260.18	78.61	80.43	77.51	2.93	6.13
78	**WT (cloned)**	WT	4	-	-	-	-	-	-	2.92	91.3	105.63	92.75	98.82	1.25	91.04	4.69	91.77	3.23	5.06	4.58	87.54	260.07	82.84	83.38	80.78	4.13	6.52

* [(0.9*A8)*100/(0.9*A8+T9)] for WT and V600E (0.9 - reduction factor for A-peak intensity)
[T12*100/(T12+G13)] for all other mutations

** operators:

C6	=IF("**C6**">5;"+";"-")
A10	=IF(("**A10**"-"**G1**")>5;"+";"-")
C11	=IF(("**C11**"-"**G1**")>5;"+";"-")
T12	=IF(("**T12**"-"**G1**")>5;"+";"-")
A18	=IF("**A18**">5;"+";"-")
G19	=IF("**G19**">9;"+";"-")
V600E/WT	=IF("**mt:wt ratio**">7;"V600E";"WT")
V600K/V600E2	=IF("**A8**"/"**T12**")>4.5;"V600E2";"V600K")

Table S3. Pyrogram sequence patterns for 36 BRAF mutations detectable by U-BRAF^{V600} assay Skorokhod et al.

	BRAF Mutation	Dispensation order																								
		2	3	4	5		6	7		8		9		10		11	12	13		14		15	16	17	18	19
		T	A	C	A		C	G		A		T		A		C	T	G		A		T	C	T	A	G
1	**p.T599del (c.1794_1796delTAC)**		mt		wt																					
	wild type	T	A	C	**A**			G				T						G	A	A	A	T	C	T	-	-
	AGTGAAATCT	-	**A**	-	-		-	G		-		T		-		-	-	G	A	A	A	T	C	T	-	-
2	**p.A598_T599insKKGNFGLA (c.1795_1796ins27)**							wt						mt												
	wild type	T	A	C	A			G				**T**						G	A	A	A	T	C	T	-	-
	TAAAAAAATAGGTG	T	7xA	-	-		-	-		-		T		**A**		-	-	G	G	-		T	-	-	-	**G**
3	**p.T599_V600>RE (c.C1796G;T1799A)**									mt		wt														
	wild type	T	A	C	A			G				**T**						G	A	A	A	T	C	T	-	-
	TAGAGAG	T	A	-	-		-	G		**A**		-		-		-	-	G		A		-	-	-	-	**G**
4	**p.T599I;V600E (c.C1796T;T1799A)**							wt						mt												
	wild type	T	A	C	A			**G**				T						G	A	A	A	T	C	T	-	-
	TATAGAG	T	A	-	-		-	-		-		T		**A**		-	-	G		A		-	-	-	-	**G**
5	**p.T599_V600>IAL (c.1796_1798>TAGCTT)**							wt						mt												
	wild type	T	A	C	A			**G**				T						G	A	A	A	T	C	T	-	**-**
	TATAGCTTTG	T	A	-	-		-	-		-		T		**A**		-	-	G		-		-	C	TTT	-	**G**
6	**p.TVKSR599_603>I (c.1796_1809>TC)**							wt								mt										
	wild type	T	A	C	A			**G**				T						G	A	A	A	T	C	T	-	-
	TATCTGGAG	T	A	-	-		-	-		-		T		-		**C**	T	G	G	A		-	-	-	-	**G**
7	**p.T599_V600insV (c.T1794_1955insGTT)**				wt									mt												
	wild type	T	A	C	**A**			G				T						G	A	A	A	T	C	T	-	**-**
	TGTTACAG	T	-	-	-		-	G		-	T	T		**A**		**C**		-		A		-	-	-	-	**G**
8	**p.T599A (c.A1795G)**											wt				mt										
	wild type	T	A	C	A			G				**T**						G	A	A	A	T	C	T		
	TGCAG	T	-	-	-		-	G		-		-		-		**C**	-	-		A		-	-	-	-	**G**
9	**p.T599_V600insT(1) (c.C1796_1797insTAC)**				wt									mt												
	wild type	T	A	C	**A**			G				T						G	A	A	A	T	C	T		
	TACTACAG	T	A	C	-		-	-		-		T		**A**		C	-	-		A		-	-	-	-	**G**

10	**p.T599T;V600E (c.A1797T;T1799A)**							wt																		mt
	wild type	T	A	C	A			G				T						G	A	A	A	T	C	T		
	TACTGAG	T	A	C	-		-	-		-		T		-		-	-	G		A		-	-	-	-	G
11	**p.T599_V600insT(2) (c.A1797_1798insACA)**						mt			mt		wt														
	wild type	T	A	C	A			G				T						G	A	A	A	T	C	T		
	TACAACAGTG	T	A	C	A	A	C	-		A		-		-		-	-	G		-		T	-	-	-	G
12	**p.T599_V600insTT (c.A1797>TACTACG)**																mt_	_wt								
	wild type	T	A	C	A			G				T						G	A	A	A	T	C	T		
	TACTACTACGG	T	A	C	-		-	-		-		T		A		C	T	-		A		-	C	-	-	GG
13	**p.V600>YM (c.G1798>TACA)**															mt		wt								
	wild type	T	A	C	A			G				T						G	A	A	A	T	C	T		
	TACATACATG	T	A	C	A		-	-		-		T		A		C	-	-		A		T	-	-	-	G
14	**p.VKS600_602>DT (c.1799_1804>ATA)**									mt				mt			mt_	_wt								
	wild type	T	A	C	A			G				T						G	A	A	A	T	C	T		
	TACAGATACTCG	T	A	C	A		-	G		A		T		A		C	T	-		-		-	C	-	-	G
15	**p.V600E(2) (c.TG1799_1800>AA)**								mt			5	:	1			mt_	_wt								
	wild type	T	A	C	A	G			-			T						G	A	A	A	T	C	T	-	-
	TACAGAAAAATCTCG	T	A	C	A	G	A	A	A	A	A	T		-		C	T	-		-		-	C	-	-	G
16	**p.V600K (c.GT1798_1799>AA)**									mt			3	:	1		mt_	_wt								
	wild type	T	A	C	A			G				T						G	A	A	A	T	C	T		
	TACAAAGAAATCTCG	T	A	C	A	A	A	G	A	A	A	T		-		C	T	-		-		-	C	-	-	G
17	**p.V600R(1) (c.GT1798_1799>AG)**									mt			3	:	1		mt_	_wt								
	wild type	T	A	C	A			G				T						G	A	A	A	T	C	T		
	TACAAGGAAATCTG	T	A	C	A	A	G	G	A	A	A	T		-		C	T	-		-		-	C	-	-	G
18	**p.V600_K601>E (c.1799_1801delTGA)**									mt			2	:	1		mt_	_wt								
	wild type	T	A	C	A			G				T						G	A	A	A	T	C	T		
	TACAGAATCTCTG	T	A	C	A		-	G	A	A		T		-		C	T	-		-		-	C	T	-	G
19	**p.V600E;K601I (c.TG1799_1800>AA;A1802T)**									mt	3	:	1	mt			mt_	_wt								
	wild type	T	A	C	A			G				T						G	A	A	A	T	C	T		
	TACAGAAATATCT	T	A	C	A		-	G	A	A	A	T		A		-	T	-		-		-	C	T	-	-

20	**p.V600D (c.TG1799_1800>AT)**									mt	1	:	3	mt			mt_	_wt								
	wild type	T	A	C	A			G				T						**G**	A	A	A	T	C	T		
	TACAGATAAATCT	T	A	C	A		-	G		A		T	A	A	A	-	T	-		-		-	C	T	-	-
21	**p.V600G (c.T1799G)**														mt	3 : 1		mt_	_wt								
	wild type	T	A	C	A			G				T						**G**	A	A	A	T	C	T		
	TACAGGGAAATCT	T	A	C	A		G	G	G	-		-	A	A	A	-	T	-		-		-	C	T	-	-
22	**p.V600E(1) (c.T1799A)**									mt		wt														
	wild type	T	A	C	A			G				**T**						G	A	A	A	T	C	T	-	-
	TACAGAGAAATCT	T	A	C	A		-	G		**A**		-		-		-	-	G	A	A	A	T	C	T	-	-
23	**p.V600M (c.G1798A)**							wt				1	:	$[I_{10} - I_4]$	*			mt								
	wild type	T	A	C	A			**G**				T						G	A	A	A	T	C	T	-	-
	TACAATGAAATCT	T	A	C	A	A	-	-		-		T		-		-	-	**G**	A	A	A	T	C	T	-	-
24	**p.V600L (c.G1978T)**							wt				1	:	$[I_{10} - I_4]$				mt								
	wild type	T	A	C	A			**G**				T						G	A	A	A	T	C	T		
	TACATTGAAATCT	T	A	C	A		-	-		-		T	T	-		-	-	**G**	A	A	A	T	C	T		-
25	**p.V600R(2) (c.AGT1797_1799>GAG)**									mt		wt														
	wild type	T	A	C	A			G				**T**						G	A	A	A	T	C	T		
	TACGAGGAAATCT	T	A	C	-		-	G		**A**		-		-	-	-	G	G	A	A	A	T	C	T	-	-
26	**p.V600A (c.T1799C)**											wt				mt										
	wild type	T	A	C	A			G				**T**						G	A	A	A	T	C	T		
	TACAGCGAAATCT	T	A	C	A		-	G		-		-		-		C	-	G	A	A	A	T	C	T	-	-
27	**p.V600K;S602S (c.GT1798_1799>AA;T1806G)**									mt						mt						wt				
	wild type	T	A	C	A			G				T						G	A	A	A	**T**	C	T		
	TACAAAGAAATCGCG	T	A	C	A	A	A	G	A	A	A	T		-		C	-	G		-		-	C	-	-	G
28	**p.K601E (c.A1801G)**							wt			$[2I_4 - I_{10}]$		:	$[I_{10} - I_4]$				mt								
	wild type	T	A	C	A			**G**				T						G	A	A	A	T	C	T		
	TACAGTGGAATCT	T	A	C	A		-	G		-		T		-	-	-	**G**	G	A	A		T	C	T	-	-
29	**p.K601N (c.A1803T)**											wt		$[2I_6 - I_{12}]$		:	$[I_{12} - I_6]$					mt				
	wild type	T	A	C	A			G				**T**						G	A	A	A	T	C	T		
	TACAGTGAATTCT	T	A	C	A		-	G		-		T		-		-	-	G	A	A	**T**	T	C	T	-	-

30	**p.K601R (c.A1802G)**																			wt					mt
	wild type	T	A	C	A		G				T						G	A	A	A	**T**	C	T		
	TACAGTGAG	T	A	C	A	-	G		-		T		-		-	-	G		A		-	-	-	-	**G**
31	**p.K601K (c.A1803G)**																			wt					mt
	wild type	T	A	C	A		G				T						G	A	A	A	**T**	C	T		
	TACAGTGAAG	T	A	C	A	-	G		-		T		-		-	-	G	A	A		-	-	-	-	**G**
32	**p.VKSRWS600_605>DV (c.1799_1814>ATGT)**								mt														wt		
	wild type	T	A	C	A		G				T						G	A	A	A	T	C	**T**		
	TACAGATGTTGGG	T	A	C	A	-	G		**A**		T		-		-	-	G		-	T	T	-	-	-	GGG
33	**p.T599_V600insDFGLAT (c.G1798_1799ins18)**							mt					1 : ⅓ I_{11}						wt						
	wild type	T	A	C	A		G				T						G	A	**A**	A	T	C	T		
	TACAGATTTTGGTCTAG	T	A	C	A	-	G	**A**	T	T	T	T	-	-	-	G	G		-		T	C	T	A	G
34	**p.K601Q (c.A1801C)**																				wt	1 : ½I_{18}		mt	
	wild type	T	A	C	A		G				T						G	A	A	A	**T**	C	T		
	TACAGTGCAA	T	A	C	A	-	G		-		T		-		-	-	G		-		-	C	-	A A	-
35	**p.VKSRWS600_605>D (c.1799_1814>A)**																						wt_	_mt	
	wild type	T	A	C	A		G				T						G	A	A	A	T	C	**T**		
	TACAGATGGGTCCCA	T	A	C	A	-	G		A		T		-		-		G G G		-		T	CCC	-	**A**	-
36	**p.VKSRWS600_605>EK (c.1799_1815>AAAAG)**																						wt_	_mt	
	wild type	T	A	C	A		G				T						G	A	A	A	T	C	**T**		
	TACAGAAAAGGGGTCCCA	T	A	C	A	-	G		A A A A		T		-		-		G G G G		-		T	CCC	-	**A**	-

* I – intensity value of correspondent dispensation order's nucleotide
dispensation order's nucleotides, which are involved into mt:wt ratio, are bolded.
recognition patterns' nucleotides are indicated in black boxes, the individual features - in grey boxes
